# Genome-Wide Fine-Scale Recombination Rate Variation in *Drosophila melanogaster*

**DOI:** 10.1371/journal.pgen.1003090

**Published:** 2012-12-20

**Authors:** Andrew H. Chan, Paul A. Jenkins, Yun S. Song

**Affiliations:** 1 Computer Science Division, University of California Berkeley, Berkeley, California, United States of America; 2 Department of Statistics, University of California Berkeley, Berkeley, California, United States of America; University of Oxford, United Kingdom

## Abstract

Estimating fine-scale recombination maps of *Drosophila* from population genomic data is a challenging problem, in particular because of the high background recombination rate. In this paper, a new computational method is developed to address this challenge. Through an extensive simulation study, it is demonstrated that the method allows more accurate inference, and exhibits greater robustness to the effects of natural selection and noise, compared to a well-used previous method developed for studying fine-scale recombination rate variation in the human genome. As an application, a genome-wide analysis of genetic variation data is performed for two *Drosophila melanogaster* populations, one from North America (Raleigh, USA) and the other from Africa (Gikongoro, Rwanda). It is shown that fine-scale recombination rate variation is widespread throughout the *D. melanogaster* genome, across all chromosomes and in both populations. At the fine-scale, a conservative, systematic search for evidence of recombination hotspots suggests the existence of a handful of putative hotspots each with at least a tenfold increase in intensity over the background rate. A wavelet analysis is carried out to compare the estimated recombination maps in the two populations and to quantify the extent to which recombination rates are conserved. In general, similarity is observed at very broad scales, but substantial differences are seen at fine scales. The average recombination rate of the X chromosome appears to be higher than that of the autosomes in both populations, and this pattern is much more pronounced in the African population than the North American population. The correlation between various genomic features—including recombination rates, diversity, divergence, GC content, gene content, and sequence quality—is examined using the wavelet analysis, and it is shown that the most notable difference between *D. melanogaster* and humans is in the correlation between recombination and diversity.

## Author Summary

Recombination is a process by which chromosomes exchange genetic material during meiosis. It is important in evolution because it provides offspring with new combinations of genes, and so estimating the rate of recombination is of fundamental importance in various population genomic inference problems. In this paper, we develop a new statistical method to enable robust estimation of fine-scale recombination maps of *Drosophila*, a genus of common fruit flies, in which the background recombination rate is high and natural selection has been prevalent. We apply our method to produce fine-scale recombination maps for a North American population and an African population of *D. melanogaster*. For both populations, we find extensive fine-scale variation in recombination rate throughout the genome. We provide a quantitative characterization of the similarities and differences between the recombination maps of the two populations; our study reveals high correlation at broad scales and low correlation at fine scales, as has been documented among human populations. We also examine the correlation between various genomic features. Furthermore, using a conservative approach, we find a handful of putative recombination “hotspot” regions with solid statistical support for a local elevation of at least 10 times the background recombination rate.

## Introduction

Recombination is a biological process of fundamental importance in population genetic inference. The crossing-over of homologous chromosomes during meiosis results in the exchange of genetic material and the formation of new haplotypes. Accurate estimates of the recombination rate in different regions of the genome help us to understand the molecular and evolutionary mechanisms of recombination, as well as a host of other important phenomena. For example, recombination rate estimates are needed in assessing the impacts of natural selection [1], [2], admixture [3], and disease associations [4].

Recombination rates have been observed to exhibit a number of interesting heterogeneities: they are known to vary in magnitude and distribution between species (e.g., [5]–[7]), between populations within species [8],[9], and between individuals within populations [9]–[12]. There is also substantial variation in different regions of the genome at different scales. At the broad-scale, for example, recombination rates in humans are known to be correlated negatively with the distance from telomeres [13], while at the fine-scale, recombination events cluster in narrow *hotspots* of 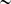
2 kb width [4], [13], [14]. In humans, hotspots are typically defined as those with statistical support in favor of at least a five-fold increase of the recombination rate [13] over the background or surrounding region, and many hotspots suggest a ten- or even hundred-fold increase. Such hotspots exhibit a powerful influence on the recombination landscape; 70–80% of recombination events in humans occur in 10% of the total sequence [8]. Extensive fine-scale variation and recombination hotspots have also been found in other species, including chimpanzees [7], *Arabidopsis thaliana* [15] and yeast [16].

The picture in *Drosophila* is however less clear. Broad-scale maps of recombination have been constructed for *D. melanogaster* by fitting a third-order polynomial to each chromosome arm [17], [18]. These give an overview of the distribution of recombination along each arm, quantifying for example earlier observations of declining recombination rates with proximity to the telomeres and centromeres. Variation on finer scales has been inferred by studies of linkage disequilibrium (LD) and by breeding experiments. Rapid and consistent decay in LD [19] leads to an absence of long haplotype blocks. There is scant evidence for hotspots either at the intensity or prevalence of those found in humans. Experimental studies of variation have produced local, fine-scale maps in *D. melanogaster* [20], *D. persimilis* [21], and *D. pseudoobscura* [22], [23], providing a resolution typically on the order of 100 kb in the regions analyzed. These experimental results suggest that regions of fine-scale variation—including some mild “hotspots” [22]—do exist in several *Drosophila* species. For example, Singh *et al.* [20] study a 1.2 Mb region of the X chromosome in *D. melanogaster*, and find 3.5-fold variation in this region, though no hotspots by the criterion mentioned above. These experimental approaches are cumbersome to recapitulate, however.

A number of crucial questions concerning *Drosophila* therefore remain unanswered. It is not known to what extent this variation is further localized to finer scales, or how common such variation is across the genome. Further, intra-specific differences in recombination rate have not been characterized. However, the advent of ambitious projects (e.g., see the Drosophila Genetic Reference Panel [18] and the Drosophila Population Genomics Project [24]) sequencing tens of *D. melanogaster* genomes each from different global populations raises the exciting prospect of addressing these and other questions. The patterns of LD in a random sample of contemporary genome sequences taken from a population contain a great deal of information regarding historical recombination events, and from these we can infer recombination rates across the genome. A number of sophisticated and computationally-intensive statistical approaches have been developed for inferring recombination rates from such data [14], [25]–[27] and for testing for the presence of recombination hotspots [28], [29], and are ostensibly suitable for this task. In particular, LDhat [14], [25], [30] is a useful software package which scales well to large datasets, and it has therefore been applied to estimating recombination rates in humans [4], [8], [13], [14], chimpanzees [7], dogs [31], yeast [16], and microbes [32], among others.

Estimating fine-scale recombination rates from recently published *D. melanogaster* genomes is, however, challenging for several reasons: First, these data exhibit a much higher density of single nucleotide polymorphisms (SNPs) than those of other species and of earlier technologies. For example, the African data considered in this paper exhibits a mean SNP rate of about 1 SNP per 38 bp for a sample of size 22, far higher than those of other recent sequencing projects (e.g., [8]). This promises an unprecedented opportunity to localize recombination rate variation to very fine scales, but making full use of these data raises further challenges in computational and statistical efficiency. Second, data generated from short-read sequencing technologies give rise to numerous missing alleles. It would be highly advantageous to be able to make use of sites in which some alleles are missing without the exponential increase in LDhat's running time that this entails. Third, the background recombination parameter in *D. melanogaster* is known to be an order of magnitude higher than in humans (the species for which LDhat's prior distributions and parameters are typically calibrated) and it is not clear how this will affect the accuracy of subsequent rate estimates. Fourth, there is a growing consensus that a considerable fraction of the genome of some *Drosophila* species is influenced by adaptive substitutions [2], [33]. Recurrent selective sweeps combined with genetic hitchhiking affect patterns of variation across many kilobases of sequence and have the potential to invalidate inferences of recombination, even leading to the possibility of spurious signals of recombination hotspots [34], [35]. By contrast, the footprints of positive selection in recent human evolution are less widespread [1]. The model underlying LDhat assumes a neutrally evolving population of constant size. While LDhat is known to be robust to mis-specification of the demographic model [14], its susceptibility to the effects of selection is less clear cut.

In this paper, we develop a new method, called LDhelmet, which addresses the above critical issues. While it employs a reversible-jump Markov Chain Monte Carlo (rjMCMC) mechanism similar to that of LDhat, our method has a number of modifications that render key advantages. Briefly, by utilizing recent theoretical advances in asymptotic sampling distributions [36]–[41], we introduce several analytic improvements to the computation of likelihoods in the underlying population genetic model, which reduce Monte Carlo errors and simultaneously provide likelihoods for all relevant samples with an arbitrary number of missing alleles. Our refinements further improve accuracy by allowing us to make full use of a quadra-allelic mutation model in which realistic mutation patterns between the four nucleotides A, C, G, T can be taken into account. Additionally, we utilize information from the available genomes of outgroup species by using them to infer a distribution on the ancestral allele at each polymorphic site in *D. melanogaster*. Taken together, our method enables us to compute fine-scale, genome-wide recombination rates with considerably improved accuracy and efficiency. LDhelmet generally produces recombination maps that are less noisy than that of LDhat's. In particular, while LDhat can infer spurious hotspots under certain types of selection, we demonstrate that our approach is much more robust.

We apply our method to data taken from two *D. melanogaster* populations, one from North America and the other from Africa, and estimate fine-scale recombination maps for each population. Then, through a wavelet analysis, we capture levels of variability and correlation of the two recombination maps, and provide a quantitative view of genome-wide inter-population comparison of recombination rates in *D. melanogaster*. We also employ the wavelet analysis to examine the correlation between various genomic features, including recombination rates, diversity, divergence, GC content, gene content, and sequence quality. At the fine-scale, we perform a conservative, systematic search for evidence of the existence of recombination hotspots and find a handful of putative hotspots each with at least a tenfold increase in intensity over the background rate. Also, we compare our recombination rate estimates with existing experimental genetic maps.

Our software LDhelmet and the fine-scale recombination maps described in this paper are publicly available at http://sourceforge.net/projects/ldhelmet/.

## Results

We applied our method to samples from two populations of *D. melanogaster*: Raleigh, USA (RAL) and Gikongoro, Rwanda (RG). The RAL dataset consisted of the genomes (Release 1.0) of 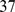 inbred lines sequenced at a coverage of 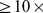 by the Drosophila Population Genomics Project [24] (DPGP, http://www.dpgp.org/). The RG dataset comprised 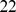 genomes (Release 2.0) from haploid embryos sequenced at a coverage of 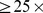 by the Drosophila Population Genomics Project 2 (DPGP2, http://www.dpgp.org/dpgp2/DPGP2.html).

### Mutation transition matrices

Using the procedure described in Materials and Methods, we were able to designate the ancestral allele in 1,755,040 of 2,475,674 high quality (quality score 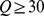) SNPs in the RAL sample (70.9%), and 2,213,312 out of 3,134,295 high quality SNPs in the RG sample (70.6%). These collections of polarized SNPs yielded the following estimates for the mutation transition matrix 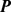, with rows and columns ordered as A, C, G, T:
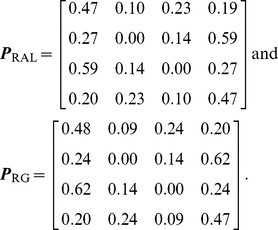
These results imply that simple diallelic models are inadequate for the *Drosophila* populations. As expected, we see a transition:transversion bias. We also observe a higher overall mutation rate away from C and G nucleotides—this pattern persists even after excluding CpG sites from our analysis (not shown). Indeed, each of the four nucleotides exhibits its own characteristic mutation distribution. There appears to be no significant difference between the transition matrices for the two populations. This is partly explained by the shared history of the two populations: There were 2,990,025 sites for which: (i) data were available in both populations, (ii) two alleles were observed in the combined sample, and (iii) one of the two alleles was assignable as ancestral. Of these, 925,569 (31.0%) were polymorphic in both populations, 800,118 (26.8%) were private to RAL, 1,262,109 (42.2%) were private to RG, and 2,229 (0.1%) were fixed differences.

For simplicity, in the analysis described in this paper, we used the same mutation transition matrix for all sites in the genome. However, we note that our method can easily handle site-specific mutation transition matrices at no extra computational cost; see Materials and Methods: for details.

### Accuracy of the method in the neutral case

To assess the accuracy of estimated recombination maps, we carried out an extensive simulation study with various simple recombination patterns, first assuming selective neutrality (the case with natural selection is discussed in the subsequent section).

The simulations assumed a finite-sites, quadra-allelic mutation model, with the mutation transition matrix 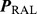 shown above and the population-scaled mutation rate 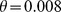 per bp. We used these transition matrix and mutation rate in LDhelmet's inference. For LDhat, we used the corresponding effective mutation rate 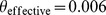 per bp (see Estimation of mutation transition matrices). Incidentally, we note that 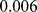 per bp is the estimated effective mutation rate for the autosomes of RAL lines [24].

**Figure 1:**
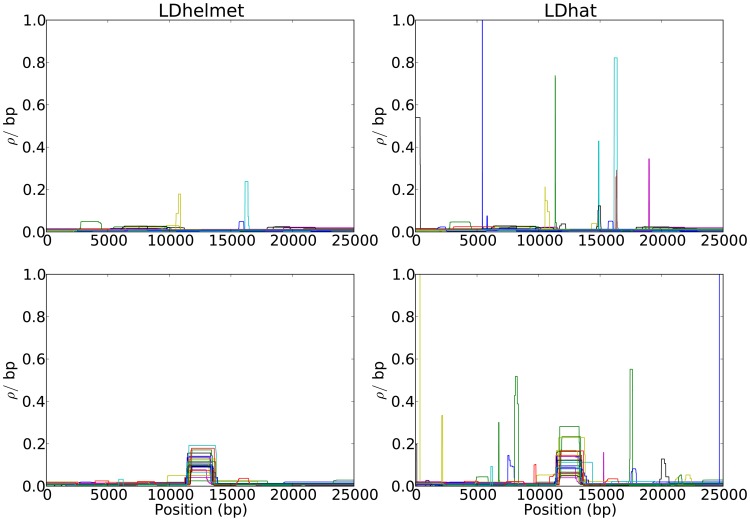
Comparison of the results of LDhelmet and LDhat for 25 datasets simulated under neutrality. In each plot, different colors represent the results for different datasets. The left and right columns show the estimated recombination maps of LDhelmet and LDhat, respectively, using the same block penalty of 50. Our method LDhelmet generally produces less noisy estimates than that produced by LDhat. (First Row) Each dataset was simulated with a constant recombination rate of 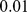 per bp. (Second Row) Each dataset was simulated with a hotspot of width 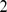 kb starting at location 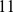 kb. The background recombination rate was 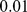 per bp, while the hotspot intensity was 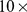 the background rate, i.e., 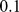 per bp. The maps are shown in their entirety, including potential edge effects.

Figure 1 shows representative examples of LDhelmet's and LDhat's results. As the figure illustrates, our method LDhelmet generally produces recombination maps that are less noisy than that of LDhat's; in particular, LDhelmet produces spurious “spikes” less frequently than does LDhat. To illustrate the impact of the spikes on the total genetic distance, the corresponding cumulative recombination maps comparing LDhelmet and LDhat are shown in Figure S1. Additional comparisons between LDhelmet and LDhat can be found in Table S1, and SNP statistics of the datasets are listed in Table S2.

In general, we observed that LDhelmet is able to identify the location of hotspots reliably. Furthermore, in the scenario considered in the second row of Figure 1, the width and height of the hotspot could be estimated very accurately; on average the total rate in the hotspot region could be estimated within 2.5% of the true value.

**Figure 2:**
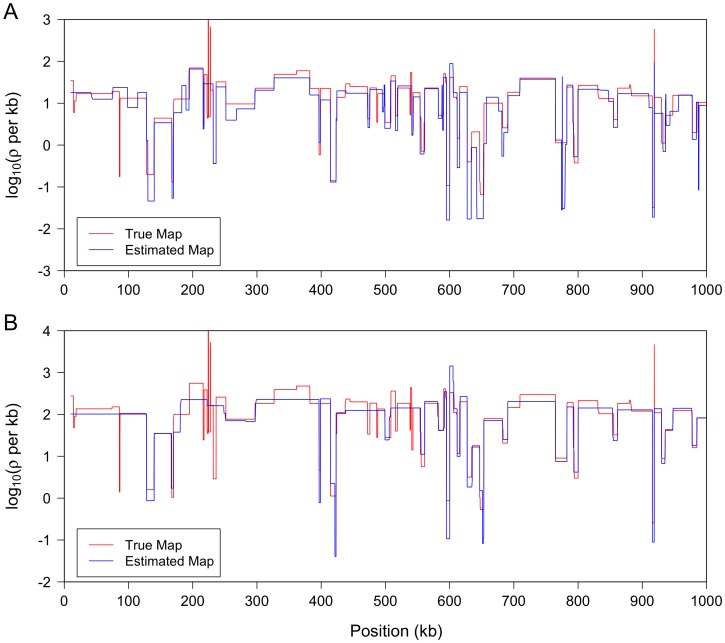
LDhelmet results on simulations with realistic variable recombination rates. In each study, the program MaCS [72] was used to simulate data, with sample size 22, for a 1 Mb region with the variable recombination map shown in red. (We used 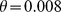; output was postprocessed to incorporate an empirical quadra-allelic mutation model.) Estimated recombination maps are shown in blue. The same block penalty of 50 was used in both cases. (A) The average recombination rate for the region is 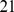 per kb, representative of the interior of the North American X. (B) The average recombination rate for the region is 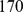 per kb, representative of the interior of the African X.

To test the performance of LDhelmet in a more realistic scenario, we simulated 1 Mb regions each with a substantial amount variation in recombination rate and with a high average rate representative of the interior of the *D. melanogaster* X chromosome. To specify realistic levels of recombination rate variability in these regions, we took as the true recombination map a 1 Mb excerpt from our estimated map for the RAL sample. To specify realistic absolute levels of recombination, we rescaled this map to match the mean (per megabase) recombination rates inferred for the X chromosomes of RAL and of RG. In Figure 2, LDhelmet's estimated recombination maps for these two scenarios are illustrated in blue, while the true maps are shown in red. These results demonstrate that, even when the average recombination rate is high, LDhelmet with our chosen block penalty in the rjMCMC is able to capture the pattern of fine-scale variation rather well. However, we note that in the top plot of Figure 2, in which case the true average rate is 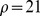 per kb, the estimated map tends to be slightly more variable than the true map. In contrast, if the true average recombination rate is substantially higher, as in the bottom plot of Figure 2 with the true average rate of 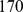 per kb but otherwise the same pattern of variation, the estimated map tends to be somewhat smoother than the true map. Clearly, there is no single block penalty value that is universally optimal in all cases, but the value we have chosen seems to yield reasonable results for *D. melanogaster* (see Materials and Methods for further details on the choice of block penalty).

### Impact of positive selection on the estimation of recombination rates

It has been previously shown [34], [35], [42] that hitchhiking can induce seemingly similar patterns of linkage disequilibrium as that created by recombination hotspots, while McVean [43] has argued that the precise signatures of selective sweeps and hotspots actually differ. To test the robustness of our method to natural selection, we simulated data under various scenarios with positive selection and recombination rate variation, and assessed the impact on our estimates of recombination rates. We generated data using a range of values for the selection strength and fixation time. See Simulation study on the impact of natural selection for details of the simulation setup.

**Figure 3:**
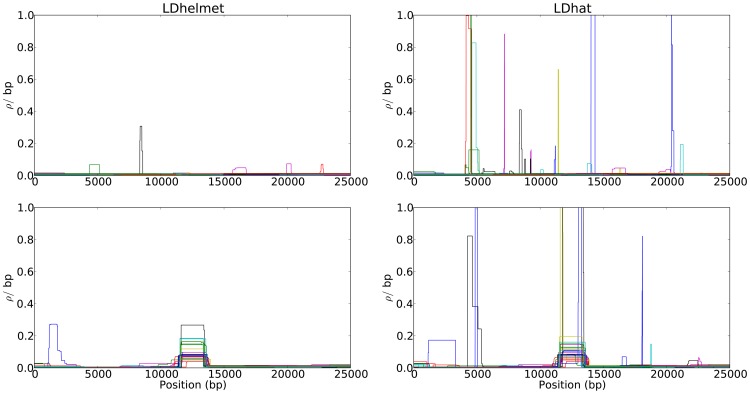
Comparison of the results of LDhelmet and LDhat for 25 datasets simulated under strong positive selection. In each plot, different colors represent the results for different datasets. The left and right columns show the estimated recombination maps of LDhelmet and LDhat, respectively, using the same block penalty of 50. In each simulation, the selected site was placed at position 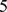 kb and the population-scaled selection coefficient was set to 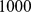. The fixation time of the selected site was 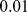 coalescent unit in the past. Although the estimated recombination maps are in general noisier than that for the neutral case (c.f., Figure 1), LDhelmet is more robust than LDhat. As illustrated in the plots, LDhat produces false inference of elevated recombination rates near the selected site more frequently than does LDhelmet. The same scenarios of recombination patterns as in Figure 1 were considered: (First Row) with a constant recombination rate of 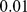 per bp, and (Second Row) with a hotspot of width 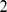 kb starting at location 11.5 kb. The background recombination rate was 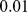 per bp, while the hotspot intensity was 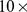 the background rate, i.e., 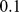 per bp. The maps are shown in their entirety, including potential edge effects.

The results of LDhelmet and LDhat for a few cases are shown in Figure 3; each plot shows the results for 25 simulated datasets illustrated in 25 different colors. The corresponding cumulative recombination maps are shown in Figure S2. For both methods, the estimated recombination maps are in general noisier than that for the neutral case (c.f., Figure 1), though LDhelmet is still more robust than LDhat. As can be seen in Figure 3, LDhat tends to produce false inference of elevated recombination rates near the selected site more frequently than does LDhelmet. A more detailed comparison is provided in Table S3 and SNP statistics of the datasets are listed in Table S2. Overall, although strong positive selection causes more noise and fluctuations in our estimates, it does not seem to produce a strong bias to the extent that would consistently lead to false inference of recombination hotspots.

The noise in our estimates of the recombination rate in the presence of selection depends on several factors. Specifically, we observed that the accuracy of our estimates decreases as the selection strength increases, whereas the accuracy improves as the distance between the selected site and the region of estimation increases. Furthermore, the more recent the time of fixation, the noisier are the estimates.

In addition to the case of a single, recent selective sweep, we also assessed the impact of recurrent selective sweeps [44], [45] on the estimation of recombination rates. Assuming that beneficial mutations fixate randomly at a given rate, we simulated three different sets of datasets with a background recombination rate of 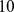 per kb, as detailed in Simulation study on the impact of natural selection. The degree to which recurrent sweeps reduce diversity in each model is summarized in Table S4. In model RS3, which has infrequent but strong sweeps, the mean number of SNPs reduces by more than a factor of 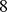 relative to the neutral model. Such a drastic drop in diversity significantly reduces the ability to perform accurate statistical inference of recombination. To infer the location of a recombination hotspot, for example, at least a few SNPs must be present in the hotspot and near its edges.

The results of recombination rate estimation under recurrent sweep models are summarized in Table 1 and Table 2. Compared to a single sweep, recurrent selective sweeps tend to decrease the accuracy of recombination rate estimates more noticeably. Furthermore, infrequent but strong selective sweeps (model RS3) have more severe impact on the accuracy than do frequent but weaker selective sweeps (model RS1). As discussed above and can be seen in Table 2, detecting recombination hotspots in model RS3 would pose a great challenge. Overall, LDhelmet generally underestimates the recombination rate in the presence of selection, suggesting that it is unlikely to produce spurious hotspots because of selection.

**Table 1:** Average recombination rates for recurrent-sweep simulations.

	No Hotspot ( 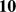 per kb)		Hotspot 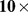 ( 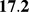 per kb)		Hotspot 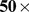 ( 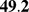 per kb)	
Model	est.	% err.	est.	% err.	est.	% err.
RS1	8.5	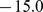	15.6	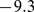	44.9	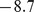
RS2	4.4	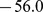	8.6	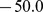	45.0	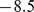
RS3	0.9	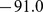	1.3	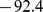	2.3	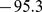
Control	9.3	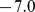	16.4	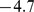	53.9	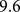

The accuracy of the recombination rate estimate for model RS3, containing infrequent but strong selective sweeps, was considerably worse than that for model RS1, containing frequent but weaker selective sweeps. The mean number of SNPs in model RS3 was a factor of 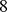 less than that in the selectively neutral “Control” model, thus reducing the ability to perform accurate statistical inference of recombination. See Simulation study on the impact of natural selection for the details of the models. For each recombination landscape, the median estimated average recombination rate is shown in the left column (“est. ”) and the percent error is shown in the right (“% err. ”). The true average recombination rate for each recombination landscape is shown in parenthesis.

**Table 2:** Hotspot areas for recurrent-sweep simulations.

	No Hotspot ( 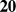 )		Hotspot 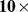 ( 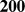 )		Hotspot 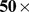 ( 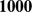 )	
Model	est.	% err.	est.	% err.	est.	% err.
RS1	16.6	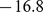	179.8	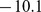	889.6	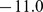
RS2	8.9	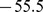	38.2	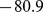	773.0	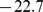
RS3	1.7	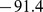	2.6	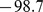	4.5	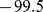
Control	18.2	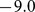	183.5	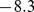	1100.0	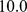

For each recombination landscape, the median estimated hotspot area is shown in the left column (“est. ”) and the percent error is shown in the right (“% err. ”). The true hotspot area for each recombination landscape is shown in parenthesis. “Control” refers to a neutral model. See Simulation study on the impact of natural selection for the details of the models and Table 1 for related results.

### Impact of demography on the estimation of recombination rates

We also tested our method on datasets simulated under a variety of demographic scenarios. Specifically, the demographic models we considered are those proposed by Haddrill *et al.* [46], and by Thornton & Andolfatto [47], comprising two exponential growth models and two bottleneck models. As in the neutral simulations, we assumed a finite-sites, quadra-allelic mutation model, with the mutation transition matrix 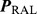 and the mutation rate 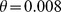 per bp. See Simulation study on the impact of demographic history for details on the other parameters used in the simulations.

Table 3 and Table 4 show the results of recombination rate estimation in this simulation study. Although the estimates are clearly less accurate compared to the case with constant population size, they are reasonably accurate in most cases. Note that the overall trend is to underestimate the true rates, in some cases only slightly.

**Table 3:** Average recombination rates for demography simulations.

	No Hotspot ( 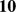 per kb)		Hotspot 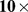 ( 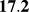 per kb)		Hotspot 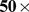 ( 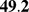 per kb)	
Model	est.	% err.	est.	% err.	est.	% err.
G1	5.8	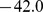	10.1	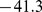	38.6	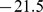
G2	7.7	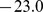	12.8	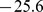	52.2	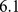
B1	7.2	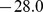	10.2	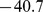	28.8	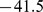
B2	1.2	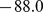	3.9	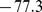	20.0	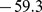
Control	9.3	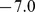	16.4	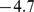	53.9	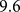

Here, “Control” refers to a neutral model with constant population size. Model B2 involved a very recent bottleneck, and we observed a reduction in diversity by about a factor of 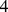 relative to the Control model. This reduction in diversity partly explains the particularly poor estimates of the recombination rate for model B2. The estimates for the other models are reasonably accurate, although they are clearly nosier compared to that for the Control model. See Simulation study on the impact of demographic history for the details of the models. For each recombination landscape, the median estimated average recombination rate is shown in the left column (“est. ”) and the percent error is shown in the right (“% err. ”). The true average recombination rate for each recombination landscape is shown in parenthesis.

**Table 4:** Hotspot areas for demography simulations.

	No Hotspot ( 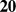 )		Hotspot 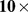 ( 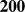 )		Hotspot 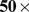 ( 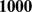 )	
Model	est.	% err.	est.	% err.	est.	% err.
G1	11.6	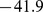	116.6	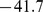	752.0	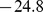
G2	15.2	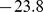	131.9	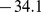	1032.6	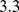
B1	14.2	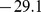	25.6	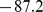	471.0	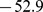
B2	1.6	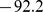	31.0	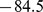	205.2	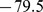
Control	18.2	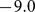	183.5	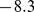	1100.0	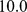

For each recombination landscape, the median estimated hotspot area is shown in the left column (“est. ”) and the percent error is shown in the right (“% err. ”). The true hotspot area for each recombination landscape is shown in parenthesis. “Control” refers to a neutral model with constant population size. See Simulation study on the impact of demographic history for the details of the models and Table 3 for related results.

As in the case of recurrent selective sweeps, demography may decrease diversity, thus hindering statistical inference of recombination. Table S4 includes the SNP statistics for the demography models we considered. In model B2, which involves a very recent bottleneck, a reduction in diversity by about a factor of 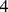 was observed, partly explaining the particularly poor estimates of the recombination rate. Table S5 shows that the average SNP density of the *D. melanogaster* data considered in this paper; note that the average SNP density of each chromosome is substantially greater than the SNP density observed in simulation model B2.

### Population-specific average recombination rates of *D. melanogaster*

The population-specific average recombination rate for each major chromosome arm is summarized in Table 5, which shows that the average rate for the African (RG) population is higher than that for the North American (RAL) population. This difference could be explained partially, but not entirely, by a difference in population size. Note that the average recombination rate in the X chromosome appears to be higher than that in the autosomes, much more so in RG than in RAL. Table 5 shows the ratio of the average recombination rate of RG to that of RAL for each chromosome arm. Although the ratio is more or less consistent for the autosomes, the ratio for the X chromosome is significantly higher. Hence, a difference in population size could explain the higher recombination rate estimates in RG for the autosomes, but it does not explain the significant increase in the recombination rate for the X chromosome of RG over RAL. Furthermore, for RAL, that the observed average recombination rate of the X chromosome is higher than that of autosomes is unexpected given that an excess of LD is observed on the X chromosome of this population [18], [24].

**Table 5:** The average recombination rate for each major chromosome arm.

	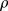 per kb			Ratio	
Chromosome arm	RAL(37)	RAL(22)	RG(22)	RG(22)∶RAL(37)	RG(22)∶RAL(22)
2L	13.3	12.4	33.2	2.5	2.7
2R	13.4	12.4	34.5	2.6	2.8
3L	13.4	12.1	44.9	3.4	3.7
3R	9.6	8.1	17.8	1.9	2.2
X	14.8	13.4	107.3	7.3	8.0

Note that RG has higher recombination rates than that of RAL. This difference could be explained partially, but not entirely, by a difference in population size. In RG, the average recombination rate of X is substantially higher than that of the autosomes. In both populations, arm 3R has a notably lower recombination rate than do the other arms. We also analyzed a smaller RAL dataset down-sampled to match the sample size of RG. The numbers in parentheses denote sample sizes.

In both populations, arm 3R has a notably reduced recombination rate compared to the other arms. This reduction is more pronounced in RG than in RAL, which could be partly explained by the fact that, in African populations, arm 3R has the largest number of common inversions [48].

To study the effect of sample size on the estimation of recombination rates, we subsampled a 2 Mb excerpt of chromosome arm 2L from each population over several repeated trials. We performed the subsampling on an excerpt rather than the entire genome for computational reasons. The averages of the estimates are shown in Table S6. Despite a slight increase in the estimate as sample size increases, the effect is small and appears to diminish with increasing sample size. We also analyzed the whole-genome RAL dataset down-sampled to match the sample size (i.e., 22) of RG. As Table 5 shows, the genome-wide average estimates produced using 22 genomes of RAL were only slightly lower than those produced using all 37 genomes. Encouragingly, our estimate (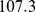 per kb) of the recombination rate for the X chromosome of RG is similar to the previous estimates for other African populations obtained using a different method: Haddrill *et al.* [46] estimated 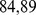, and 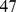 per kb for the X recombination rate in three African populations.

To assess the effect of SNP density, we thinned the SNPs on chromosome arm 2L and chromosome X of the RG dataset to the corresponding SNP densities of RAL, and performed inference on the resulting data. The results summarized in Table S7 show that although SNP density indeed influences the ability to estimate recombination rates, the impact is not nearly large enough to account for the difference between the observed recombination rates of RAL and RG on the X chromosome.

Finally, as there exist several inversions in *D. melanogaster*, we analyzed regions of inversion excluding individuals known to carry the inversion [49]. The comparison of excluding individuals with inversions and the original analysis is shown in Table S8. Note that for each inversion, only a small number of individuals carry it. We found that excluding the individuals with inversions did not significantly affect the recombination rate estimates.

### Comparison with experimental genetic maps

**Figure 4:**
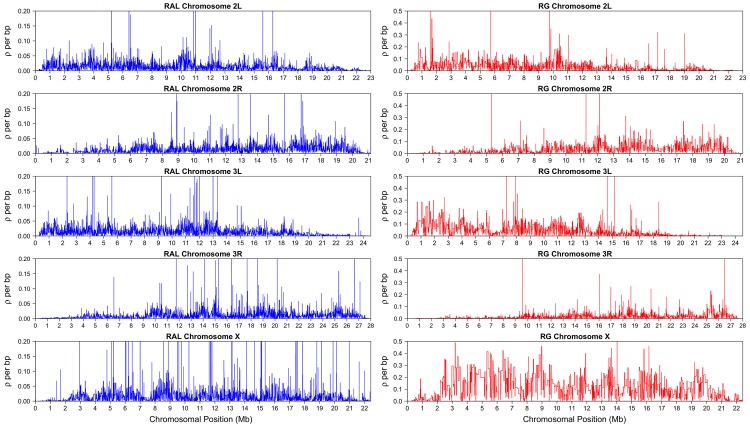
LDhelmet's estimated fine-scale recombination maps for RAL and RG populations of *D. melanogaster*. The North American sample (RAL) comprised 37 genomes, while the African sample (RG) comprised 22 genomes.

LDhelmet's fine-scale recombination maps for RAL and RG are illustrated in Figure 4; files containing the corresponding numerical values are publicly available. To assess the accuracy of our recombination estimates obtained via statistical analysis of population genetic variation data, we compared them to genetic maps obtained experimentally.

**Figure 5:**
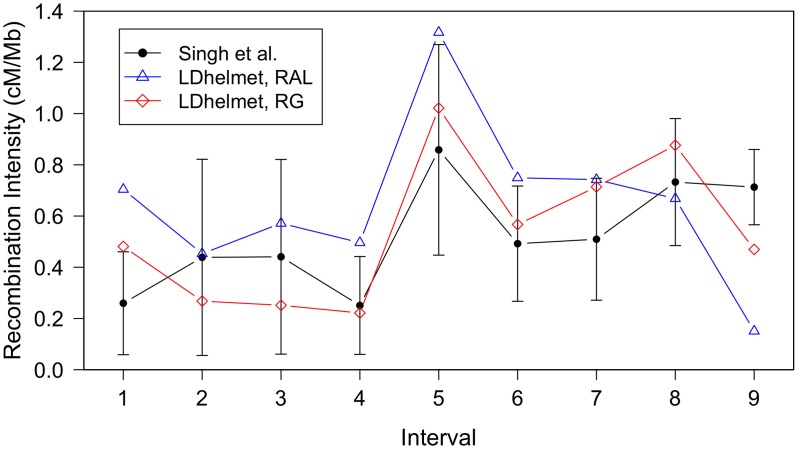
Comparison of LDhelmet estimates to the empirical genetic map of Singh *et al*. The experimental genetic map of Singh *et al.* [20] is shown in black with 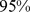 confidence intervals. The LDhelmet estimate for the RAL sample is shown in blue, while the estimate for the RG sample is shown in red. The LDhelmet estimates were converted into units of cM/Mb by normalizing them to have the same total genetic distance as the empirical map for the region. The three maps demonstrate high correlation, especially near the center of the region, where they share the highest peak in the same interval.

Singh *et al.* [20] examined the fine-scale recombination rate variation over a 1.2 Mb region of the *D. melanogaster* X chromosome using a genetic mapping approach, by crossing an African line with a line presumably of North American origin (a cross between two lines from Bloomington Drosophila Stock Center). For their experiment, Singh *et al.* genotyped 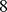 SNPs and identified two flanking genes, *white* and *echinus*, with visible phenotypes. They found statistically significant heterogeneity in this region, with differences in rate up to 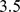-fold. In Figure 5, estimates from LDhelmet for both the RAL and RG samples are shown, along with the genetic map from [20]. Both estimates from LDhelmet mostly fall within the 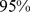 confidence intervals of the empirical estimate, with the exception of the outermost intervals. The three maps share the same overall shape, including the location of the highest peak. We find 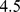-fold variation in the RG estimate, which is comparable to the 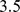-fold variation obtained by Singh *et al.* The high correlation among the three maps give us confidence that our estimates from the statistical analysis of population genetic data accurately represent the true underlying recombination map.

**Figure 6:**
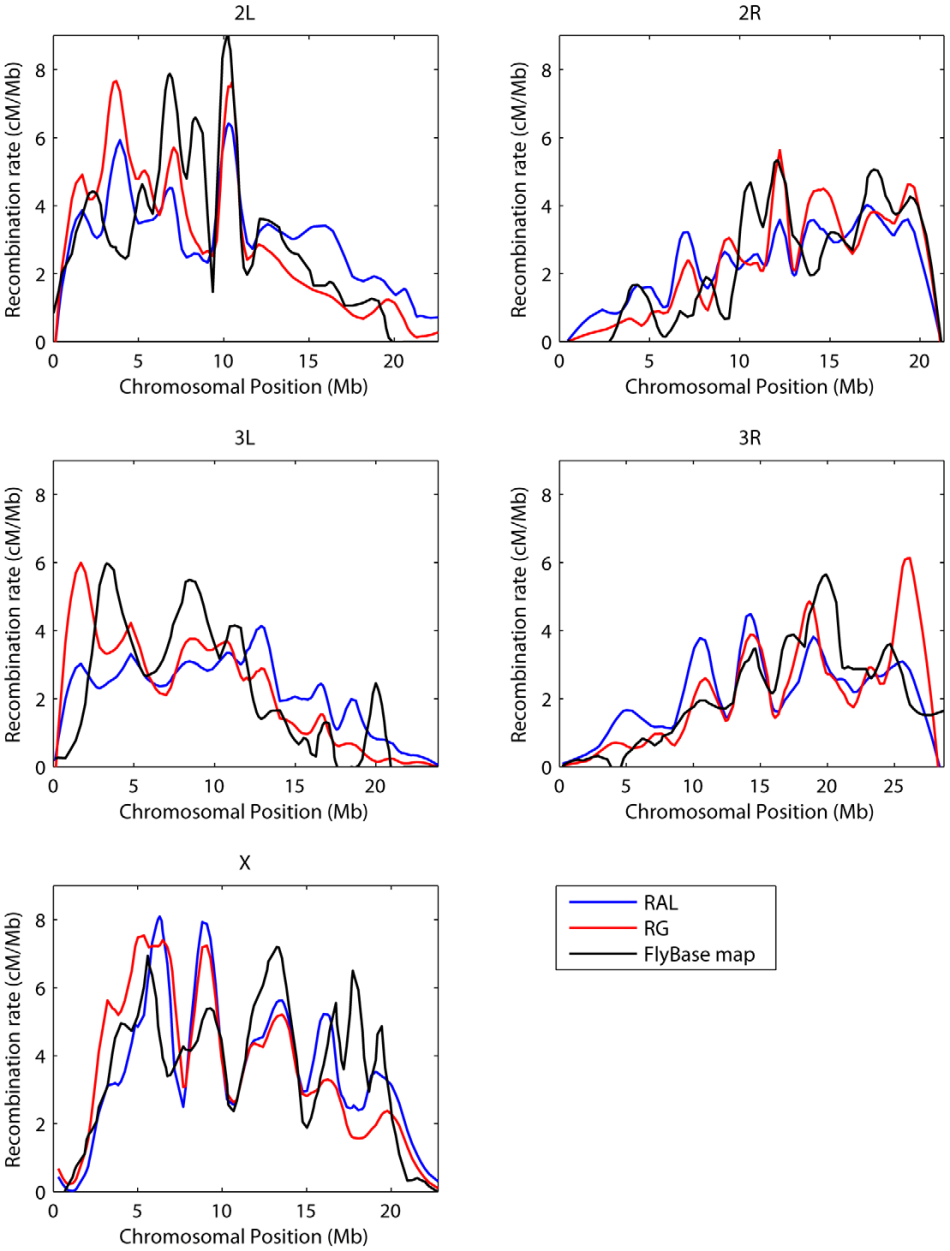
Comparison with FlyBase genetic map. Plotted for each chromosome arm are the estimated recombination maps using our method and the consensus experimental map hosted at FlyBase [50]. To ease comparison each map is LOESS-smoothed using a span of 15%. LDhelmet estimates were converted into units of cM/Mb by normalizing them to have the same total genetic distance as the empirical map.

In a second study, we compared our chromosome-wide recombination estimates with a consensus genetic map for each chromosome arm based on data hosted at the FlyBase website (http://www.flybase.org [50]). To facilitate a comparison with this map, resolution of which is roughly 200 kb, we binned our data into the same cytogenetic subdivisions [24] and LOESS-smoothed the results, with a span of 15%; a correspondingly LOESS-smoothed version of the FlyBase data was kindly provided to us by C.H. Langley. A comparison of the maps is shown in Figure 6; evidently, the three estimates show broad agreement, each capturing key features such as the spike in recombination near position 10 Mb on arm 2L, as well as a series of dramatic changes in recombination rate across chromosome X. When the recombination map for RAL is regressed on the FlyBase maps, the coefficient of determination, or proportion of variability explained by the simple linear regression model, is 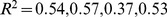 and 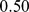 for chromosome arms 2L, 2R, 3L, 3R, and X, respectively; the corresponding values for RG are 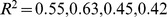, and 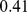. These correlations are lower than those seen in a comparison of statistically- versus experimentally-derived maps in humans (e.g. 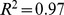 [13]), though in that case the experimental data from pedigrees were of higher quality. As noted by Langley *et al.* [24], data on which the FlyBase map is based is highly edited and based on heterogeneous experimental conditions with sometimes conflicting results.

### Recombination hotspots

As discussed in the sec:introduction, it is well known that in humans and many other eukaryotes recombination tends to cluster in recombination hotspots, regions of approximately 2 kb wide in which the rate of recombination may be one or more orders of magnitude higher than the background rate [4], [12]–[14]. However, it is an open question whether hotspots exist in the *D. melanogaster* genome, or to what extent recombination rates vary on a fine scale.

**Figure 7:**
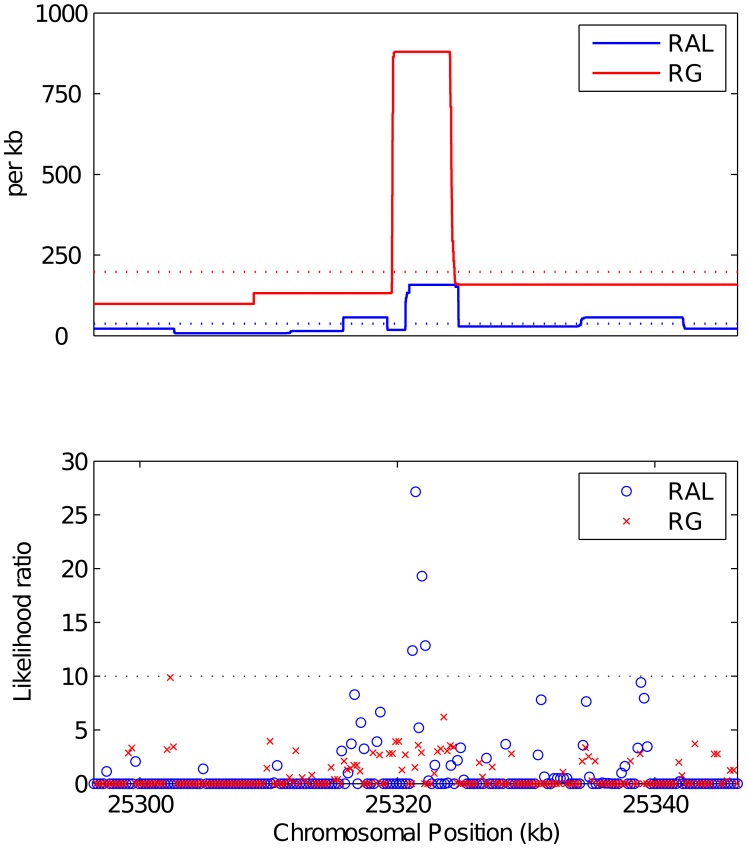
A putative hotspot found by LDhelmet and confirmed by sequenceLDhot. (Top): Estimated recombination rate for RAL (blue) and RG (red) in a 50 kb region of chromosome 3R, and their respective mean recombination rates in this region (dotted). (Bottom): Evidence of recombination hotspots in the same region, evaluated according to sequenceLDhot. The dotted line shows the likelihood ratio cutoff we used.

We first searched for the most extreme forms of recombination rate variation—namely, recombination hotspots. Using a highly conservative approach described in Materials and Methods, we initially identified nineteen and five putative autosomal recombination hotspots from the RAL and RG data, respectively. Of these, respectively six and four were also detected by the hotspot detection software sequenceLDhot [29]. These ten hotspots, the width of which ranges between 0.5 kb and 6.8 kb, are listed in Table 6. All were found in genic regions, with all except one overlapping exons and one contained within an intron. An example of a RAL hotspot is shown in Figure 7, where we also show the RG recombination map. The fine-scale recombination maps in this region for the two populations are clearly highly correlated, with both RAL and RG exhibiting a tenfold increase in recombination rate within almost identical 4 kb intervals, though only the hotspot of RAL was also found by sequenceLDhot. We note that the power of sequenceLDhot may be further reduced by the apparent preference (not shown) for putative hotspots to reside in regions in which the “local” background rate is higher than that of the chromosome arm as a whole. In light of these factors, it is likely that several more hotspots would have been found in one or both populations under a more relaxed definition, though it is clear that they are far scarcer, and less hot, than in humans.

**Table 6:** Putative recombination hotspots in *D. melanogaster* found by our method.

Dataset	Arm	Gene	Start	End	Width (kb)	#SNPs	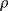 per kb	Ratio to armwide mean
6*RAL	2L	CR43314	11966311	11966880	0.6	20	140.8	11
	3L	CG9384, CG17173	14759823	14761142	1.3	30	177.9	13
	3R	Cys	10394533	10395940	1.4	42	100.8	10
	3R	CG7530	10552022	10553677	1.7	65	110.6	11
	3R	Ccap	18526587	18527115	0.5	23	122.1	13
	3R	CG2010, Trc8	25320629	25324745	4.1	169	154.9	16
4*RG	2R	DJ-1 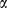 , AGO1	9830014	9830946	0.9	53	547.3	14
	2R	CG15706, Tsf3	12109706	12116536	6.8	344	545.2	14
	2R	CG4927, CG8317	12460329	12466422	6.1	255	431.4	11
	3R	nAcR 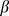 -96A	20339494	20340164	0.7	33	219.7	12

These putative hotspots were confirmed by the hotspot detection software sequenceLDhot [29].

### Genome-wide fine-scale recombination rate variability

It is apparent from both RAL and RG maps shown in Figure 4 that recombination rates vary on multiple scales, from the very fine to the very broad, as has been observed in a number of other species [7], [13]–[16]. It is clear, for example, that recombination rates tail off towards each end of the arm, with the reduction towards the telomere much more precipitous than the pericentromeric reduction. The latter reduction is evident from as far as the start of heterochromatic sequence a few megabases from the centromere, in agreement with other broad-scale estimates of recombination [17], [18], although we do not find a complete absence of recombination here.

**Figure 8:**
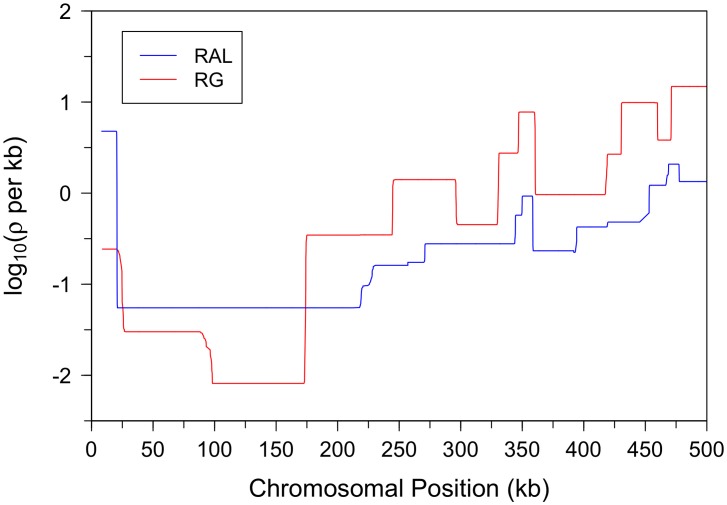
Fine-scale recombination maps for the X chromosome subtelomeric region. The telomere is at the left end of the region. The recombination rate between positions 10 kb and 20 kb is considerably higher than the rate in the subtelomeric region immediately to the right. This trend is much more pronounced in the North American X than in the African X, consistent with a previous study [51].

Figure 8 shows that the recombination rate in the X chromosome between positions 10 kb and 20 kb is noticeably higher than the rate in the subtelomeric region to the right. This trend is much more pronounced in the North American X than in the African X, consistent with a previous study by Anderson *et al.* [51]. The telomere-associated sequence (TAS), located to the left of position 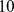 kb, was not available in our data, but Anderson *et al.* provided evidence that the TAS region in the North American X exhibits even higher recombination rate than that in the subtelomeric region between positions 10 kb and 20 kb.

As shown in Figure 4, the largest difference between the estimated recombination maps of the two populations is observed in the X chromosome. First, the recombination map in the African X is generally much higher than that in the North American X. Second, there is noticeably less variation in the estimated African X recombination map. As mentioned earlier in the discussion of our simulation study, when the average recombination rate is as high as that of the African X, the amount of variation in our estimated map tends to be somewhat lower than the true variation. Hence, the observed reduction in variation could be partially attributed to our method being not sensitive enough in that range of very high rates. More generally, it is also true that Fisher's information for data on sequence variation is lower in regions of high recombination (), which could create an inherent limitation in our ability to infer recombination rate changes here.

#### Recombination around transcription start sites

To assess the pattern of recombination around genes, we plotted the average recombination rate as a function of distance from the transcription start sites (TSS). As shown in Figure S4, the plots for RAL and RG show high similarity in shape, despite differences between their fine-scale recombination maps. Also, note that the plots follow a similar pattern as in human [4], [12], [13], chimpanzee [7], and mouse [52], although the gene density of *D. melanogaster* is much higher than that of the other species.

#### A wavelet analysis

To carry out a more methodical analysis of recombination rate variation within and between the two populations, and its correlation with other genomic features, we performed a wavelet analysis (Materials and Methods). Wavelet analyses are suitable for detecting localized, intermittent periodicities embedded in the data, across a range of possible scales. Our inputs are two sets of discrete “time”-series data representing the recombination maps of RAL and RG, binned into a recombination rate in each 250 bp window. Each is transformed into a collection of coefficients indexed by position (“time”) and scale, and describe the variation in the input signal at each position and scale. The scale index may be discrete or continuous, and in this paper we make use of both types of transform as appropriate. Although the wavelet transform may be complex-valued, it can be summarized by a plot of its *(local) power*: the square of the norm of the wavelet coefficients at each position and scale. Taking the mean power across all positions yields the *(global) wavelet power spectrum*, which summarizes how the total variability in the signal is explained by heterogeneity at different scales. Further, a correlation between the wavelet coefficients from two different “time”-series datasets can identify how a change in one signal predicts a change in the other, at a given scale. One advantage of the wavelet approach is that one does not have to choose the appropriate window size in advance, which is important since analyses of genomic data on different pre-chosen scales can give conflicting results (e.g., [2], [23], [53]).

**Figure 9:**
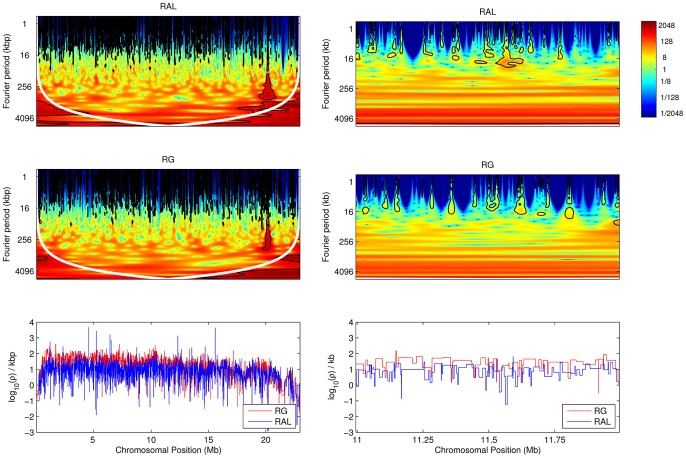
Local wavelet power spectrum of recombination rate variation across chromosome arm 2L. The whole arm is shown on the left, and a detailed (central) 1 Mb is shown on the right, for RAL and RG. Black contours denote regions of significant power at the 5% level, and the white contour denotes the cone of influence. Color scale is relative to a white noise process with the same variance. Lower panels show estimates of the corresponding recombination maps.

To illustrate, continuous wavelet transforms of the recombination maps of chromosome arm 2L are shown in Figure 9; wavelet transforms for the rest of the genome are shown in Figures S5, S6, S7, S8. For brevity we focus on chromosome arm 2L throughout; results for the remaining arms are given in the Supporting Information. We can interpret these transforms with reference to the wavelet transform of a constant recombination map, which would yield essentially zero power (dark blue) everywhere. Clearly the transform is highly inconsistent with a constant map. Regions of high power, shown at the red end of the spectrum and corresponding to wavelet coefficients of large magnitude, are consistent with variation in recombination rate at the given location (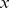-axis) and at the given scale (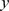-axis). Intuitively, a location of high local power in the wavelet transform suggests that a useful proportion of the variability in our dataset is well-explained if we track it by placing a wavelet function at this position and with the appropriate width corresponding to this scale. One way to evaluate the most significant regions of the time-frequency domain is to compare the transformed data with the transform of a null first-order autoregressive process with the same variance; thus, we allow for some variability as we scan along the data from left to right, and identify those regions (black contours in the figures) with wavelet power significantly above the null expectation.

Observe that highest power (red color) is seen in Figure 9 at the broadest scales (long periods) and at very fine scales. The former reflects the centromeric and telomeric decline in recombination rate, and we see that the centromeric decline has a more pronounced effect on the largest periods (though we caution that these signals are below the *cone of influence*, a region whose wavelet transform may be unduly distorted by edge effects [54]). Analogous patterns are evident in the other chromosome arms (Figures S5, S6, S7, S8). Notice also that very fine-scale variation is manifested in high power regions at small periods (e.g., Figure 9, right-hand plots). While there exists some previous evidence for localized fine-scale variation in recombination rate in *D. melanogaster* [20], our finding that it is widespread across the genome is novel.

#### Correlation of the two recombination maps at various scales

**Figure 10:**
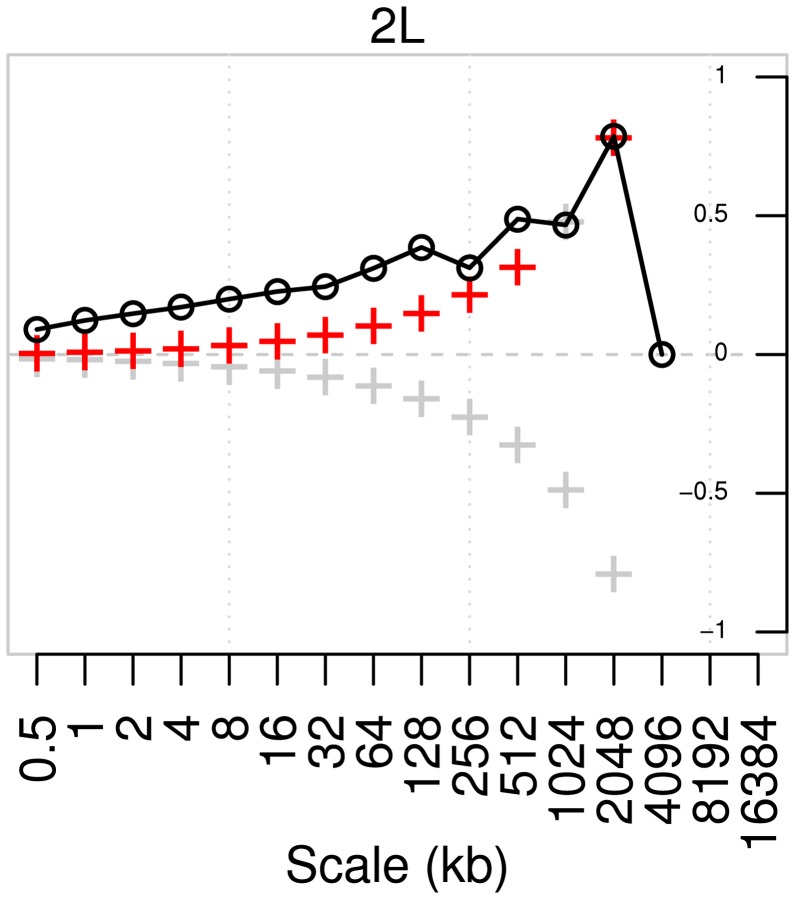
Pairwise correlation of detail wavelet coefficients of RAL and RG recombination maps for chromosome arm 2L. Black circles denote Kendall's rank correlation between pairs of detail coefficients at each scale. Crosses denote the correlation that would be required for significance at the 1% level in a two-tailed test; red crosses are those scales at which the correlation is in fact significant.

Although there is some correlation in fine-scale variation between the two populations (for example, its lower volatility in region 11.2–11.25 Mb of arm 2L; see the right column of Figure 9), it is far from strong. To explore how well correlated the two maps are at each scale, we computed the pairwise correlations between wavelet coefficients of the two maps, after applying a discrete (Haar) wavelet transform following [53] (Figure 10, Figure S9). This choice of transform decomposes a dataset into a series of wavelet coefficients for each of a discrete set of scales. The decomposition provides a series of *detail* coefficients measuring changes between neighboring observations, and a series of *smooth* coefficients which provides a smooth approximation of the original signal [55]. The correlation, at a given scale, between the detail coefficients of the wavelet transform of two maps can then be computed, and those with significantly high correlation identify the scales at which the two maps do co-vary. Across all arms and across all except the broadest scales there is a highly significant correlation in the variability of the two maps (Kendall's rank correlation, two-tailed test at 1% significance). The lack of correlation at broader scales is probably due to lack of power: for example, at the 1% level there are too few data points for this test to have any power at any scale broader than 4 Mb.

**Figure 11:**
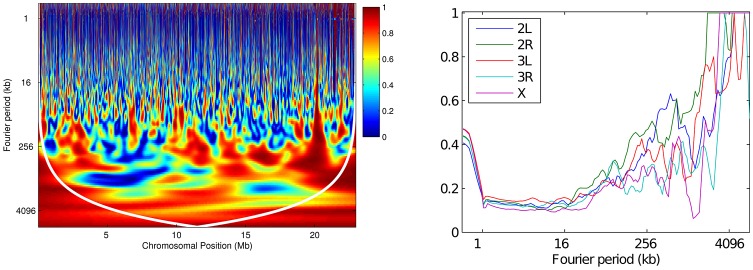
Wavelet coherence analysis comparing RAL against RG. (Left): Wavelet coherence of the two maps for chromosome arm 2L. The cone of influence is shown in white. (Right): For each arm, the plot shows the fraction of the genome with significantly high coherence at the 5% level, at each scale.

Given the similarities between the two populations, it is perhaps not surprising that their recombination rates are highly correlated when assessed globally. To further elucidate how this correlation varies in different regions of the genome, we performed a *wavelet coherence* analysis (see sec:method), which can be regarded informally as calculating a squared correlation coefficient between the variation of the two maps at each position as well as at each scale. Wavelet coherence analysis thus evaluates correlations in local, rather than global, power. Results are shown in Figure 11 and Figure S10. It is clear that the correlation between the two maps is found nonuniformly along the chromosome. While there is high correlation at all positions at the broadest (megabase) scales, at smaller scales there exist regions of very low correlation, even when the overall correlation between the two maps at this scale is high. For example, the average coherence between the two maps at the 256 kb scale is 0.59 over the whole of 2L, compared to only 0.19 in the region 5–6 Mb. (Note that the persistently high correlation seen near position 20 Mb across many scales, reflects a particular region of missing data in both populations, and hence flat recombination.) Although the existence of regions of low coherence is partly explained by statistical error (Figure S11), it does not explain the drop fully. Thus, at least some isolated regions of low correlation are consistent with the idea that biological differences between the two populations create local differences in the recombination rate.

### Correlation of recombination rates with other genomic features

**Figure 12:**
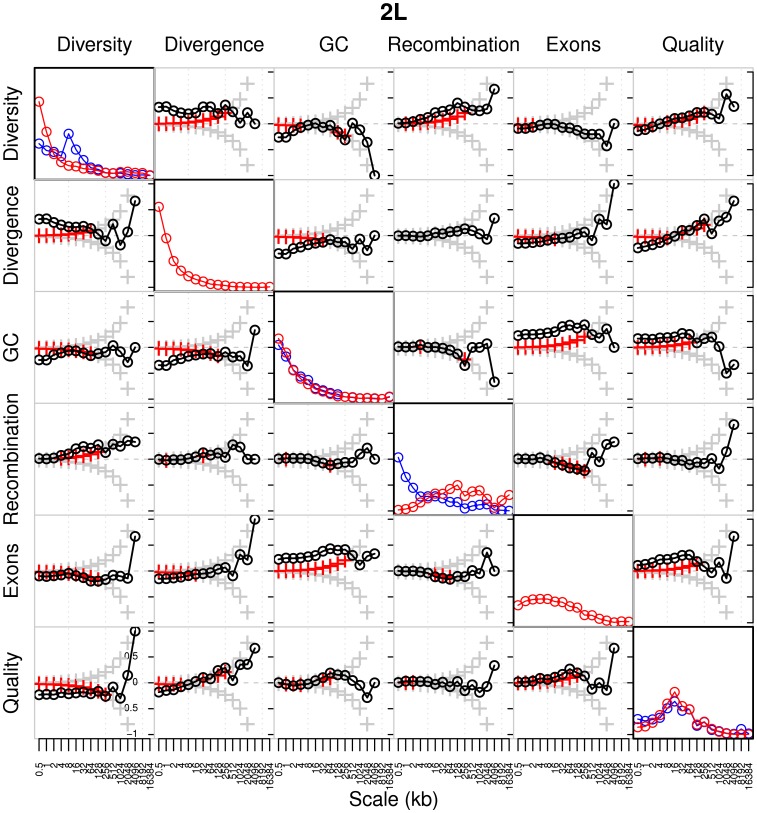
Global wavelet power spectrum and pairwise correlations of detail wavelet coefficients of RAL and RG data for chromosome arm 2L. Diagonal plots show the global wavelet power spectrum of each feature of the RAL (blue) and RG (red) data. Off-diagonal plots show Kendall's rank correlation between pairs of detail coefficients at each scale, with respect to the wavelet decomposition of the two indicated features. Crosses denote the correlation that would be required for significance at the 1% level in a two-tailed test; red crosses are those scales at which the correlation is in fact significant. The bottom left and top right plots correspond to RAL and RG, respectively.

The use of wavelets enables us to compare how changes in the rate of recombination along the genome correlate with other genomic features. For each population we computed pairwise correlations between the detail coefficients of the following features: diversity (mean fraction of pairwise differences between each individual in the population, within sequenced nucleotides), divergence (fraction of differences between the reference sequences of *D. melanogaster* and *D. simulans*), GC content, gene content (fraction of sites annotated as exonic), and sequence quality (Phred score), as well as the recombination rate, with each feature measured in 250 bp windows (see Materials and Methods). Results are shown in Figure 12 and Figure S12, and follow a similar analysis performed by Spencer *et al.* [53] on human data. From these results we can make a number of observations detailed below.

#### The power spectra of each genomic feature

As in humans, we find the greatest heterogeneities in divergence and GC content at the finest scales, and in gene content at intermediate scales. Heterogeneity in diversity and recombination are strikingly different when we compare RAL and RG: recombination shows the greatest heterogeneity at fine scales in RAL and at intermediate scales in RG (as in humans); the reverse is true of diversity. These patterns are broadly repeated for each arm (Figure S12), although it should be noted that the lack of heterogeneity in recombination at fine scales in the RG data may partly be a consequence of its high background recombination rate leading to lower resolution (as discussed above; see Figure S3). Limitations such as these notwithstanding, the broad agreement between chromosome arms gives ground for optimism that the signals are not swamped by noise.

#### Pairwise covariation of genomic features

The off-diagonal plots in Figure 12 provide a great deal of information about the covariation of several pairs of genomic features. Some are predictable and also found in humans [53]. For example, there is a strong positive correlation between diversity and divergence at fine and intermediate scales, consistent with variation in mutation rates at different positions in the genome. As a second example, both the negative correlation between gene content and diversity and the negative correlation between gene content and divergence are predicted by the observation that exons tend to be under greater selective constraint.

Perhaps the most notable difference between *D. melanogaster* and humans is seen when we examine the correlation between recombination and diversity. In humans this correlation is weak and extends only up to approximately the 4 kb scale. Spencer *et al.* [53] therefore infer that the influence of recombination on changes in diversity is primarily local in nature and driven by recombination hotspots. In *D. melanogaster*—for both the RAL and RG data—the positive correlation between recombination and diversity is stronger and acts up to intermediate scales, approximately 2–256 kb. Interestingly, the correlation at very fine scales, 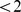 kb, is weaker and for some chromosome arms nonsignificant (see Figure S12). These findings suggest both that a local influence of recombination hotspots on diversity is weaker or absent in *D. melanogaster*, consistent with the paucity of hotspots found in our search described above, and that some other phenomenon exerts an effect on diversity, but not divergence, over much larger scales. Clearly, one candidate is the action of selection, whose impact on the correlation between recombination and diversity is well appreciated [20], [21], [23], [24], [56], [57]. The scale up to which we have been able to detect this correlation, around 256 kb (with some differences according to the population and chromosome arm examined), is surprisingly large given that the footprints of selective sweeps are typically in the region of up to 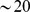 kb [2], [24].

Finally, it is notable that there is a significant negative correlation between the recombination rate and gene content at intermediate scales, in both RAL and RG and across all chromosome arms (though the signal is weaker on the X chromosome). This is consistent with the apparent preference for crossovers to occur outside exonic sequence [58], although we note that the effect does not appear to act at the finest scales—recall also that all but one of the putative hotspots identified in Table 6 do in fact overlap with exonic sequence.

#### A linear model analysis

**Figure 13:**
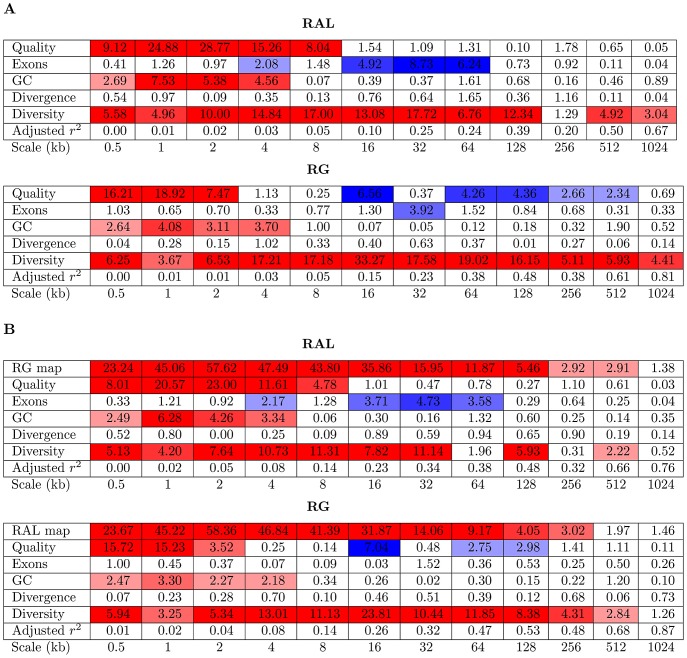
Linear model for wavelet transform of recombination map of chromosome arm 2L. (A) In a linear model for the detail coefficients of the wavelet transform of the recombination map of chromosome arm 2L, covariates are the detail coefficients of wavelet transforms of data quality, gene content, GC content, divergence, and diversity. Shown is the −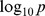-value of the regression coefficient at the given scale, as determined by a t-test. Colored boxes indicate significant relationships, with red positive and blue negative. Also shown in the adjusted 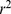. (B) As above, but with the recombination map of the other population as an additional covariate.

Given the strong but imperfect correlation between the recombination maps of RAL and RG, can we use the same genomic features to predict the regions in which the two maps might differ? To extend the analysis above and to address this question, we used a linear model analysis of the wavelet coefficients of each recombination map, using wavelet coefficients of other features as predictors. This analysis is similar to that described in [53], though their interest was in the prediction of changes in diversity rather than recombination. For each population and at each scale, we fit a linear model for the detail coefficients of the recombination map using as predictors the detail coefficients of wavelet transforms of sequence quality, gene content, GC content, divergence, and diversity (Figure 13A; Figures S13A, S14A, S15A, S16A). We find changes in diversity to be a strong predictor of changes in recombination across all chromosome arms and across many scales, though the effect is on some arms somewhat weaker (and nonsignificant) at the finest scales. Again, this is in contrast to the primarily local relationship between changes in diversity and recombination in humans. In addition to diversity, there are additional positive influences of GC content and sequence quality at fine scales; a weak negative influence of gene content at intermediate scales; and, in RG only, a negative influence of sequence quality at broad scales. Each of these signals is much weaker on the X chromosome (Figure S16), except the influence of diversity as a predictor of recombination, which still extends up to the megabase scale despite much higher absolute rates of recombination on this chromosome. The positive association between GC content and recombination is consistent with biased gene conversion [21], [53] and/or codon bias [21], [22], though we note an apparent negative correlation between GC content and recombination at broader scales (Figure 12, Figure S12).

When the recombination map from the other population is added as an additional covariate, it is the strongest predictor of recombination rate at all but the broadest scales (Figure 13B; Figure S13B, S14B, S15B, S16B). Of the remaining covariates, those which were previously highly significant predictors now generally have reduced impact. However, their 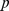-values at several scales are still highly significant, indicating that they offer explanatory power of the recombination rate over and above that provided by the recombination map of the other population. In particular, diversity remains a strong positive predictor of levels of recombination over most scales.

## Discussion

We have developed a new method, LDhelmet, which is able to provide accurate estimates of recombination rates using genomic data from *D. melanogaster*. Although our focus has been on this species, the features of our method should offer improvements in the estimation of recombination in other species too. For example, the desire to efficiently incorporate sites in which some alleles are missing is a common issue when data are generated by next-generation sequencing technologies. We believe that our method will find many further applications in other datasets.

Using our method, we have performed a genome-wide comparison of fine-scale recombination rates between two populations of *D. melanogaster*, one from Raleigh, USA (labeled RAL) and the other from Gikongoro, Rwanda (labeled RG). While earlier studies have largely been confined to regions of moderate resolution, we find extensive fine-scale variation across all chromosomes and in both populations. A notable difference between the two recombination maps is the higher overall recombination rate in RG than in RAL. Our method estimates the composite parameter 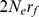, where 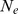 is the effective population size and 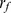 is the (female) rate of recombination per generation, so this difference is partly explained by a difference in effective population size. However, further differences between chromosomes—namely, the inflated recombination rates in the X chromosome relative to autosomes—lead us to invoke biological differences too, particularly the role of polymorphic inversions. There may also be other, unappreciated, biological factors causing an increase in 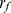 on the X chromosome.

In addition to the higher absolute rate of recombination in RG, a further difference between the populations merits discussion: the relative increase in recombination on the X chromosome compared to the autosomes is much more pronounced in RG than in RAL. In the African population, estimates of the ratio 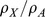 lie in the range 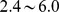, whereas in the North American population they lie in the range 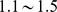 (Table 5). There are several possible explanations for the difference between the two populations. First, RAL may have experienced a historical population bottleneck. The effect of a population bottleneck on LD is stronger on the X chromosome than on the autosomes [59] (a similar effect on diversity is also seen [60]). Thus, a population bottleneck leads to an increase in LD on the X chromosome over and above the increase on the autosomes. A bottleneck in the non-African population is a sensible proposition since *D. melanogaster* is a human commensal of African origin which has colonized North America more recently. Bottlenecks in non-African populations of *D. melanogaster* have been inferred from genetic data by others [46], [47]. Furthermore, as shown in our simulation study, bottlenecks tend to cause our method to underestimate the true recombination rate, so the bottleneck explanation would be consistent with the fact that our recombination rate estimates for RAL are lower than that for RG. Second, the impact of polymorphic inversions may be greater in RG, since the African population has a high frequency of polymorphic inversions in the autosomes and in the centromere-proximal X. The observed increase in the recombination rate in the African X could be partially attributed to *interchromosomal effect* [61], [62]. A third possible explanation is the more efficient role of selection on the X chromosome when nonneutral mutations are recessive: such mutations can more easily be exposed to the action of selection in their hemizygous state in males. This effect will be more pronounced in RAL if it has undergone greater selective pressures, as seems likely in its adaptation to a new environment. Unraveling the relative importance of these possible explanations merits further investigation.

At fine-scales, we also find extensive differences between the recombination maps of the two populations, for which a simple difference in effective population size is not a sufficient explanation. Wavelet coherence analysis reveals high correlation at broad scales but regions of low correlation at fine scales, as has been documented among human populations, and in comparison between humans and chimpanzees [8], [9]. The advantage of a wavelet coherence approach is that it further identifies the locations of similarities and differences. However, the causes of these differences remain to be understood. One noteworthy result of our analysis is that changes in diversity are a strong positive predictor of changes in recombination in one population, even when the recombination map of the other population is included as a covariate. A possible explanation for this observation is that the two populations have undergone separate selective sweeps, with sufficient impact on the genome that the correlation between recombination and diversity can still be detected even when the recombination map of the other population is used as a covariate. We note that a partial overlap in the signature of selective sweeps was also found by Langley *et al.* [24]. Using a metric based on valleys of diversity, they found that 44% of diversity valleys in RAL overlapped with those found in an African sample. There are of course other possible explanations for the observed correlations between diversity and recombination; it is known that background selection—the loss of neutral diversity due to linked *deleterious* mutations—can also induce such a correlation (see Charlesworth [63], [64] and references therein). The relative importance of these types of selection in distinguishing the two populations is obviously deserving of further study.

Access to a fine-scale map lets us address a crucial question of the distribution of recombination in *Drosophila*: whether they localize into recombination hotspots. Using a conservative approach, we found a few regions with solid statistical support for a local elevation of at least 10 times the background recombination rate (Table 6). With the caveat that we used a high block penalty in the rjMCMC and employed a stringent hotspot detection strategy, overall our findings support the belief that extreme localization of recombination into hotspots is not prevalent in *D. melanogaster*; in humans, on the other hand, the list of well-supported hotspots exceeds 30,000 [4], many of which exhibit much more than a tenfold increase and have a common mechanism for recruiting the recombination machinery [6], [10], [11]. Singh *et al.* [20] therefore reserve the term “recombination peaks” for the milder variability they find, and it could be the case that what we have found in this paper are the most extreme examples of these peaks. Having said that, we also note that, as discussed earlier in our simulation study, the ability to perform accurate statistical inference of recombination (in particular, detecting hotspots) gets significantly reduced when recurrent strong selective sweeps are in play. It is hence possible that there are actually more hotspots in the *D. melanogaster* genome than our study could find.

We have focused on estimating and characterizing the recombination map itself and on its correlation with a set of important genomic annotations, but given such a map one can tackle many further problems. The question of primary sequence influences of recombination localization can now be addressed with much greater power. In humans, the 13 bp motif CCNCCNTNNCCNC has been found to be over-represented in hotspots, consistent with its recruitment of the protein PRDM9 which has been implicated in the hotspot usage [6], [11]. Searches for motifs in *Drosophila* that correlate with fine-scale recombination rate have been undertaken in *D. pseudoobscura* [22], [23], *D. persimilis* [21], and *D. melanogaster* [58]. Motifs that correlate with fine-scale recombination in humans are also significant in some of these species [21], [23], which is unexpected given the rapid turnover of motif usage in humans and chimpanzees [6]. In a recent pedigree study, Miller *et al.* [58] were able to localize with high precision fifteen crossover events on the X chromosome of *D. melanogaster*. From these they identified the 7 bp motif GTGGAAA as significantly enriched in the vicinity of these crossovers. Further study is required to validate this motif and to search for others, and our maps should prove useful in this regard.

Finally, our work should be of interest since a fine-scale recombination map is a prerequisite of studies seeking to estimate the influence of natural selection on the genome [1]; those lacking such a map retain this caveat [2]. Although these inferences of recombination and selection rely on the same data and have the potential to distort each other, it is reassuring that our method is robust to the influence of positive selection, and that it shows good agreement with existing experimental estimates of recombination. In our simulation studies we focused on the effects of hard sweeps, since they are thought to be an important mode of adaptation in *Drosophila* [2], [33], [45] and are expected to have the strongest effect on patterns of variation. Aside from additional noise resulting from a reduction in diversity, there is little bias introduced by failing to include selection in the assumed model, at least under the parameters we considered. This is consistent with the observation that a recurrent sweep model does not have a striking effect on LD beyond that predicted by the reduction in diversity [59]. Nonetheless, further investigation is warranted on the effects of other types of selection, and on the development of methods that can account for recombination and selection jointly.

## Materials and Methods

### Data

The mean coverage of the RAL data was 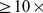. Regions of residual heterozygosity and regions of identity-by-descent between genomes were masked in the RAL data, in addition to a quality filter of Q30 applied to both populations. Preliminary analysis by the DPGP2 group found evidence of admixture among 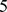 of the 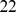 RG lines we considered, in addition to evidence for minor levels of identity-by-descent between genomes. To maintain a reasonable sample size, these regions were not masked in the results presented in this paper. We did repeat several of our analyses with these regions excluded and generally found little difference. Despite the extensive filtering, which increases the amount of missing data, the runtime complexity of our method does not increase from a lack of data, as it does for LDhat.

The data were divided into overlapping blocks of 4,400 SNPs each, with 200 SNPs of overlap on either end of a block. For every block, LDhelmet was run for 3,000,000 iterations after 300,000 iterations of burn-in. The map for each chromosome or chromosome arm was constructed by removing 200 SNPs from the ends of the blocks and concatenating the blocks together.

### Population-scaled recombination parameter

The aim of our method is to infer the fine-scale map of the population-scaled recombination rate in *D. melanogaster*, in which recombination occurs only in females. The population-scaled recombination rate between a pair of sites in the 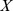 chromosome is defined as 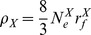, where 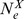 is the effective population size for X and 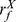 is the probability of recombination between the sites per generation per X chromosome in females. The population-scaled recombination rate between a pair of sites in an autosome is defined as 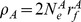, where 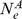 is the effective population size for the autosome and 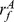 is the recombination rate between the sites per generation per autosome in females. Furthermore, 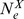 and 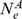 are defined as 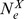 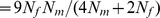 and 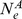 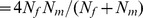, where 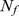 and 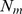 denote the effective number of female and male individuals in the population. If we assume 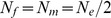, we obtain 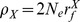 and 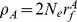.

In contrast to recombination, mutation occurs in both males and females. We denote the X chromosome mutation rates in females and males as 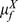 and 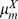, respectively, and the autosomal mutation rates in females 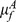 and 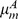 males as and, respectively. Then, the population-scaled mutation rates for X and the autosomes are given by 
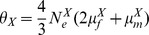
 and 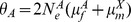, respectively. Further, if 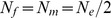, then the expressions simplify to 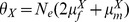 and 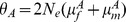.

In our statistical model, we allow the recombination rate to vary across the genome. We use to denote generically the population-scaled recombination map, which is a function of genomic position. For ease of notation, we do not add a subscript to 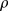 to distinguish between X and autosome; it should be clear from the context which is intended. Similarly, we use 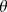 to denote generically the population-scaled mutation rate.

Our objective is to estimate the recombination map from population genomic DNA sequence data. Our approach introduces several key improvements to the method LDhat [14], [30] (v2.1 used throughout), which was first developed for estimating fine-scale recombination maps in humans. Below is a brief description of LDhat, followed by the details of our improved method LDhelmet.

### A brief description of LDhat

Given a sample of chromosomes from a population, LDhat estimates the recombination map 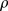 within a Bayesian setting, placing a prior on the map. To avoid overfitting, 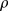 is assumed to be a step function (i.e., a piecewise constant function). The prior is a distribution on the number of times 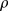 changes value, the locations of such changes, and the value of each piecewise constant segment. LDhat employs reversible-jump MCMC (rjMCMC) [65] to sample from a posterior distribution over a sample space of step functions where different parts of the space have different numbers of parameters.

Denote the likelihood of 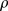 and 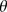 by 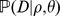, where 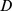 represents a set of phased haplotypes. Rather than compute the full likelihood, which is in general intractable except for a very small sample, LDhat computes an approximation known as the *pairwise composite likelihood* [30], [66]. For every pair of SNPs in a short region, the pairwise likelihood is computed under the coalescent with recombination, and the product over all such pairwise likelihoods serves as an approximation to the full likelihood. This approach scales well to large datasets, and has been demonstrated through simulation studies to provide a reasonable approximation to the full likelihood [30].

The two-locus likelihoods are precomputed and stored in a lookup table for computational efficiency. In LDhat, the two-locus likelihoods for sample configurations with no missing data are precomputed by importance sampling [67]. Then, the likelihoods for sample configurations with missing data are computed (and stored) as they are encountered during data analysis, by marginalizing over appropriate configurations with no missing data; the running time of this procedure is exponential in the number of missing entries in the configuration.

There is one likelihood table for every choice of mutation parameter 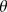, and likelihoods are precomputed over a grid of the recombination parameter 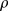. In LDhat, the default is over 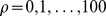 and a two-allele model is assumed, with mutation transition matrix ***P*** 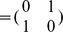 at each site. That is, when a mutation event occurs, the allele changes to the other type with probability 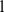.

### An overview of our new method LDhelmet

To accommodate the higher recombination rate observed in *D. melanogaster*, we introduce several key modifications to LDhat to improve the accuracy and robustness of recombination map estimation. These modifications are summarized as follows:

1. Instead of using importance sampling to compute the two-locus likelihoods, we compute them by solving a systems of recursion relations, thereby producing more accurate lookup tables. An additional benefit of this approach is that we can handle large amounts of missing data at no additional computational cost, since the likelihoods of configurations with missing data naturally appear in the system of recursions.
2. Our method incorporates a general quadra-allelic mutation model, whereas LDhat assumes a diallelic model. As a consequence, we can handle complex mutation patterns between the A, C, G, T nucleotides. Furthermore, our method can use different mutation transition matrices for different sites at no extra computational cost.
3. We make use of the recent work [36]–[39] on asymptotic sampling distributions to incorporate a larger range of 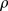 values in the lookup table in a computationally tractable manner.
4. The lookup table exhibits a finer grid resolution for values of 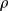 in regions of higher likelihood curvature, for improved accuracy.
5. We infer a distribution on the ancestral allele at each site and use this information to compute more refined likelihoods.
6. The prior for the recombination map is more flexible and can be tailored to the particular species under analysis. For example, when analyzing a species that is believed to have significantly higher recombination rates than that of humans, as is the case for *D. melanogaster*, one should not use the same prior as for humans.

The computational tractability of the first two modifications listed above is dependent on the following approximation we employ: We assume that each site underwent at most one mutation in the entire genealogical history of the sample. This assumption is reasonable for small values of 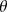, as is the case for *D. melanogaster*, and it provides several computational advantages, described in the following sections.

### Two-locus recursion relation

For generating two-locus likelihood lookup tables, we replace importance sampling with solving recursion relations [68] (see also [36]–[39], [69]). These recursion relations necessitate the solution of large systems of equations in the possible observed sample configurations. However, the one-mutation-per-site assumption leads to gains in efficiency that make such systems soluble. To illustrate, consider first a random sample drawn from a single locus. We use the notation 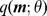 to denote the probability that a sample of 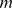 alleles taken at random from the population in some fixed order leads to the one-locus configuration ***m*** 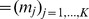, where 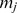 is the number of samples with allele 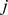; if we are modeling, say, the evolution of DNA nucleotides, then 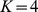 and 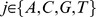. (It is implicit that this probability is also a function of the mutation transition matrix 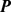 at this locus.) It is well known (e.g., [70]) that 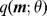 satisfies
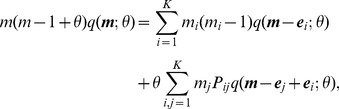
(1)for which a closed-form solution is not known in general. Here, 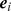 denotes a unit vector with 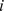
th entry 1 and the rest zero. In a later section, we describe a method for using outgroup data to infer which of the alleles in our samples is ancestral. When the identity of the ancestral allele (i.e., the allele of the most recent common ancestor of the sample) is presumed known, say type *a*, the appropriate boundary condition for use with (1) is
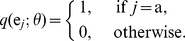
As an alternative to working with (1), we can seek a solution for the joint probability of obtaining the configuration 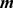 with the event that it arose as the result of precisely 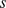 mutation events in the history of the sample, a probability we denote by 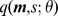. Then we have [70]:
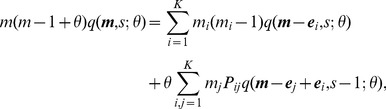
(2)with
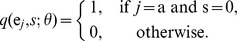
The advantage of the one-mutation-per-site assumption is then apparent: 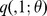 is known in closed-form [40], [41]:
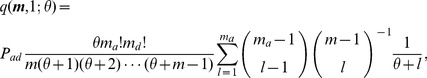
(3)where the only nonzero entries of 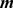 are 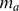 and 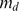, corresponding to a sample comprising 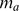 copies of the ancestral allele type 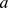 and 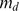 copies of a derived allele type 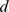. Hence, in this case we entirely circumvent the need for a numerical solution to a large system of linear equations. Provided the mutation rate per site is sufficiently small, the error 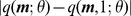 should be negligible.

We can make similar gains in a two-locus model by reducing a large system of equations to a much smaller system, albeit one that still requires a numerical solution. The idea is similar to that described above, though notation is more complicated: the precise form of the system is provided in Text S1. In the present paper, the largest sample size we work with is 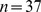. This leads to a very large system of equations that must be solved: Accounting for symmetries, the total number of complete configurations of size 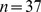 is approximately 1,300. When we count all configurations encountered in the RAL data—including those with missing alleles—this number rises to 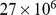. In the two locus case, the quantity of interest is 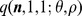, the probability of obtaining the two-locus configuration 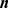 together with the events that there was precisely one mutation event at each of the two loci. Here, 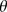 denotes the mutation rate and 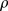 denotes the recombination rate between the two loci. Provided we work with the reduced system of equations for 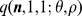 as outlined above, it becomes feasible to solve the system for every sample of size 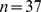, and thus to generate *exactly* solved lookup tables for later use. Table S9 shows the running time of this recursion-based likelihood computation as a function of sample size 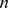.

### Missing data

Because the two-locus recursion relation is solved jointly for every configuration, this also gives us exact solutions for every subconfiguration at no extra computational cost. In particular, we emphasize that we also obtain likelihoods for all relevant configurations with any *missing* data, at no extra computational cost. By contrast, when LDhat encounters a configuration in which some alleles are missing, its approach is to marginalize over missing alleles by summing over the relevant entries in its lookup table for fully-specified haplotypes, but the time required for this computation is exponential in the number of missing alleles. The extent of missing data in the *D. melanogaster* genomes is such that this approach is impracticable. On the data we analyzed, we masked all alleles with a quality less than 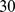. For the RAL lines, about 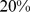 of the data was missing, and for the RG lines, about 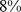 of the data was missing. The more missing data there is, the more expensive marginalization becomes, and the greater the number of distinct configurations present in the data.

### Incorporating a quadra-allelic mutation model

One key advantage of our approach is that it can make use of all four alleles (A, C, G, T) in sequence data, together with the ancestral alleles inferred from outgroup sequences. This is achieved with modifications to the boundary conditions of the appropriate two-locus recursion described above. In combination with the one-mutation-per-site assumption, this allows us to use a full 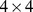 transition matrix 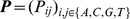 to model realistic mutation patterns between nucleotides, with no significant amount of extra computation: Suppose the ancestral allele at each of a given pair of segregating sites is known to be 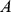 and 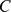, respectively. At the first site some chromosomes exhibit a derived 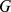 allele, and at the second site some chromosomes exhibit a derived 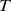 allele. Because of the one-mutation-per-site assumption and the decoupling of the genealogical and mutational processes under neutrality, it is easy to see that the likelihood of this two-locus configuration has a dependence on 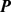 only through a single multiplicative factor 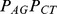. Hence, this expression can be factorized completely out of the two-locus likelihoods and hence from our lookup tables. The remaining quantity, which represents the probability of observing a particular configuration up to the identities of the alleles involved, can be multiplied by the relevant pair of entries in 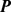 for *any* observed combination of nucleotides. To be precise, if 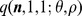 denotes our solution to the system of equations described above, this argument shows we can write
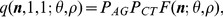
(4)for some function 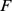 independent of 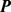. [The single-locus analogue of this result is evident in equation eq:one-locus-solution.] We then need to store only 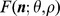. If later we see the same combination of haplotype counts but for a different combination of nucleotides, we can reuse this quantity and multiply it by different relevant entries in 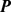. For simplicity, in our analysis we used the same for each site in the genome, but note that, because of the factorization in (4), it is possible to use different mutation transition matrices for different sites at no extra computational cost.

This approach easily generalizes to the case where the ancestral allele is not known or where we only have a distribution on the ancestral allele at each site. We can simply take the weighted average over each of the four possible combinations of ancestral alleles, weighted with respect to their distributions. In the case where no information is known about the ancestral alleles, this reduces to using the stationary distribution of 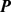 as the distribution over ancestral alleles at each site.

### Estimation of mutation transition matrices

Because we are now able to make full use of a quadra-allelic mutation model, we developed a method to estimate the 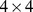 mutation transition matrix 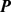 from empirical data, for subsequent use in our recombination rate inference. We use the following parsimony-based method to estimate 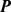 by inferring the ancestral allele at each site in *D. melanogaster* by comparison with aligned outgroup reference genomes of *D. simulans*, *D. erecta*, and *D. yakuba*. We designate the ancestral allele at each dimorphic site in *D. melanogaster* using the following rule. If the alleles of the three outgroups are not all missing at this site and together exhibit precisely one of the four possible nucleotides, and if this allele agrees with one of the two observed in *D. melanogaster*, then this is designated as the ancestral allele. Otherwise, it is considered unknown and discarded from the analysis. (We also discarded triallelic and quadra-allelic sites.) A related approach is used in the Drosophila Population Genomics Project in the estimation of divergence. We tried both more and less restrictive parsimony rules, as well as excluding CpG sites from our analysis; neither variation substantially altered our results.

Given a large collection of SNPs in our dataset for which the ancestral allele is known, we can infer the identities of the alleles involved in the mutation event at each polymorphic site. For example, an A/G polymorphism with A ancestral implies a historical A
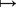
G transition. The relative frequencies of each type of event, normalized to account for varying genomic content of the four nucleotides, determines our empirical estimate of 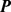. To be precise, let 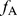 denote the total number of A nucleotides in the *D. melanogaster* genome, of which 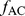, 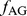, and 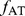 have been inferred to be A
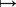
C, A
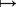
G, and A
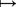
T polymorphisms, respectively. (For consistency we restrict all these definitions only to those monomorphic or dimorphic sites for which sufficient, consistent outgroup information is also available, as required above.) We make analogous definitions for 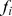 and 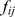, for each 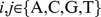. Finally, let 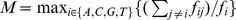, the largest empirical frequency of mutation away from any particular nucleotide. The appropriate choice for 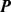 is given by
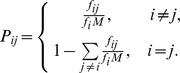
Division by 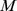 ensures that, without loss of generality, one entry in the diagonal of 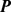 is zero. By allowing the diagonal entries of 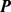 to be nonzero, different nucleotides can have different overall mutation rates. The total “effective” mutation rate—that is, mutations not involving the diagonal entries of 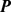
—is calibrated against classical infinite-sites-based estimators: for RAL this is 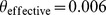 per bp (autosomes) and 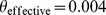 per bp (X chromosome). For RG we used 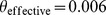 per bp for all chromosomes. Since we are to use a general quadra-allelic model in which both effective and ineffective mutations are permitted to occur, the appropriate choice of 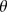 for use with 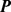 is such that it exhibits the same overall rate of effective mutations:
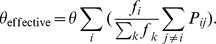



### Ancestral allele distribution

When it is not known which of the two alleles at a polymorphic site is ancestral, one can use the stationary distribution of 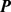 as a prior distribution over the ancestral allele. However, when additional information is available, such as sequence data from an outgroup, we can use the information to update our prior beliefs about the identity of the ancestral allele, thus allowing a more accurate estimate of the recombination map. In our application, we used the *D. simulans* outgroup information to update our prior distributions on the ancestral alleles of the *D. melanogaster* samples. Specifically, for each *D. melanogaster* genome, we used the software psmc [71] to estimate, at each site, a distribution on the time to the most recent ancestor (TMRCA) of the *D. melanogaster* and *D. simulans* genomes. Given the TMRCA, we integrate over possible mutations occurring according to 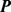 along the two branches, to obtain a distribution on the ancestral allele. Finally, for each site, we aggregate each of these pairwise distributions into a single distribution on the ancestral allele, and use this distribution in the computation of our likelihoods. Further details are provided in Text S1.

### Padé approximants

Recall that LDhat's lookup tables are precomputed over a grid: 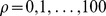. For a pair of sites with a recombination rate greater than 100, the likelihood at 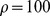 is used as an approximation. This can create systematic errors in the likelihood [36]. Instead, for 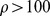 we compute accurate approximations to the two-locus likelihood using the method of Padé summation described in Jenkins & Song [38]. Briefly, one Taylor expands 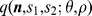 about 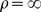 and uses the method of Jenkins & Song to compute the first few terms in the expansion. In practice this Taylor series rapidly diverges for values of 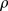 of interest, but it can be made into an accurate, convergent approximation of 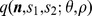 by replacing this truncated series with a rational function approximation whose own Taylor series agrees as far as possible, a technique known as Padé summation. We modified the analysis of Jenkins & Song to account for our new system of equations described above (see Text S1). We precompute 11 Padé coefficients (up to 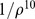 in the Taylor series expansion of the likelihood about 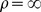) for every sample configuration of size 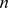, which gives an extremely accurate approximation for every 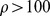 (not just integral values). Usually, the “join” between the Padé approximant for 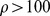 and the true likelihood for 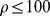 is indistinguishable. We also employ a “defect heuristic” [38] with threshold parameter 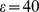 to correct for potential effects from singularities in the Padé approximants. As in the direct computation of the likelihoods from the system of equations, obtaining the Padé coefficients for a given configuration also yields the coefficients for all its subconfigurations. This approach is therefore well-suited to data with a large proportion of missing data. Table S9 shows the running time for the Padé coefficient computation as a function of sample size 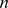.

### Lookup table grid resolution

One can imagine that it would be useful to have a more refined lookup table in regions of higher curvature of the likelihood. In such regions simply using integral values of 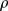 might be too coarse. Since the lookup tables will be used for every conceivable pairwise dataset, we should be interested in the expected curvature of the likelihood curve at 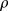, across datasets drawn under a model with the same 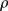. (That is, the curvature at some 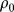 is most important for datasets that we are likely to see when the recombination rate really is 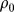.) This is reflected by Fisher's information:
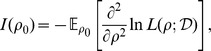
which can be estimated from an existing lookup table using the second-order central difference operator. As is evident from Figure S3, curvatures are generally higher in the range 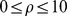, and so we changed the increment between 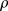 values in the lookup table from 1 to 0.1 in this range.

### Prior on recombination map and block penalty choice

LDhelmet places a prior distribution on the number of change points, the positions of the change points, and the heights of the change points in the recombination map. The prior on the number of change points is, as in LDhat, a Poisson distribution with mean equal to 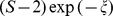, where 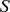 is the number of SNPs in the data and 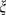 is a user-defined parameter called the *block penalty*. The positions of the change points are distributed uniformly, and the distribution on the heights of the change points is user-settable as exponential, gamma or log-normal.

One should be mindful that LDhat was designed for background recombination rates an order of magnitude less than that used in the simulations. In particular, LDhat implements the exponential prior but the mean is hard-coded for human data. Adjusting the mean of the prior according to the expected background recombination rate is necessary to obtain meaningful results. For example, using a prior suitable for humans on *Drosophila*-type data produces poor estimates with little to none of the true variation in the underlying recombination map (simulations not shown). To facilitate a comparison, we modified the source code of LDhat such that its prior was similar to the one used by LDhelmet. Without such modifications, the estimates from LDhat were not comparable to LDhelmet's estimates. In the simulations and analysis, we used an exponential prior with the mean adjusted for the expected background rate of *D. melanogaster*.

The block penalty controls the extent of variation in the estimated recombination map. In general, the higher the block penalty, the smoother the estimated map. We carried out a simulation study to choose a conservative penalty value to reduce false positive inference of hotspots, at the expense of tolerating more false negatives.

In this simulation study, we considered the following three scenarios: no recombination variation (constant rate), moderate variation (with a hotspot of width 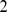 kb and intensity 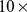 the background rate), and high variation (with a hotspot of width 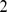 kb and intensity of 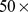 the background rate, such as that seen in humans). We simulated 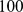 datasets of each kind, with a fixed background rate of 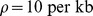 in all cases.

After considering a variety of evaluation metrics for measuring the accuracy of an estimated map, we found the 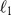-distance between the true map and the estimated map to be the simplest to interpret and assess, where the 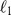-distance is the sum of the point-wise differences between the true and estimated maps. For the three scenarios described above, Figure S17 shows the average 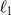-distances between the true recombination maps and the estimated maps for various block penalty values and recombination landscapes. For each dataset, we ran LDhelmet for 250,000 iterations after a 50,000 iteration burn-in. We observed that noise from overfitting is reduced for higher block penalties. Based on our simulation study, we chose a conservative block penalty of 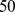 in our analysis of the real data.

In our simulation study for evaluating the choice of block penalty on realistic data (Figure 2), we used the program MaCS [72] to simulate a 1 Mb region with a highly variable recombination map. (We used 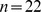 and 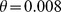; output was postprocessed to incorporate an empirical quadra-allelic mutation model.) The map's variability was taken from a 1 Mb excerpt of the estimated recombination map of the X chromosome for the RAL sample. The total recombination rate for the region was then rescaled to match the mean (per Mb) rate of the RAL X chromosome (to create a “RAL-like” map) or the RG X chromosome (to create a “RG-like” map; see Figure 2).

### Simulation study on the impact of natural selection

In order to simulate datasets that had been affected by natural selection, we focused on modeling the effects of sites experiencing positive, genic selection, i.e. selective sweeps. We investigated two modeling scenarios: First, the effect of a single, strong sweep with its strength, fixation time, and location treated as fixed parameters. Under some parameter combinations, we expect such sweeps to substantially reduce observed polymorphism levels. Second, we considered data generated under the influence of a recurrent sweep model, in which the ages and genomic locations of sweeps occur randomly. In this scenario, we chose the parameters of the model (selection coefficient and rate of fixation of beneficial mutations) such that expected polymorphism levels were concordant with observations in *D. melanogaster*. While the second scenario is likely to be a more realistic model for the forces affecting variation in *D. melanogaster* genomes, its inherent randomness introduces additional noise. The first scenario allows us to study the effects of a sweep with particular characteristics under a controlled environment.

Under both scenarios, we again simulated data under three possible recombination landscapes: a flat recombination rate of 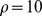 per kb except for a 2 kb-wide hotspot at the center of the sequence, of relative strength 1 (no hotspot), 10, or 50; we also post-processed all outputs to allow for a full quadra-allelic mutation model, using the mutation transition matrix 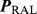. To reconstruct the recombination maps of simulated data, we used the following parameters for LDhelmet and LDhat: 250,000 iterations after 50,000 iterations of burn-in for LDhelmet, and 1,000,000 iterations after 100,000 iterations of burn-in for LDhat. We chose the number of iterations such that the two methods would require about the same computational time.

#### Single sweep model

In order to simulate datasets that had experienced a single hard sweep, we used the software mbs [73]. Using mbs, we simulated the trajectory of a selected allele backwards from its fixation time at the present back to the random time of its birth, then post-processed the software's output to translate the trajectory such that its fixation time was instead at time 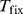 in the past. (This lets us condition on 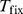, which is otherwise not possible using the software.) Subject to this trajectory we then used mbs to simulate 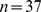 samples of 25 kb of sequence in the vicinity of the selected site. We simulated 100 trajectories for each possible combination of the following choices of parameter: selection strength 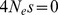, 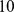, 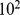, 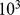, and 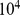 (where 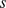 is the relative fitness); 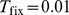, 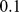, 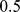, 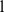, and 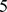, in units of 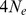 generations; and three possible recombination landscapes (see above). For each trajectory we simulated, independently, a 25 kb sample with the selected site at coordinate 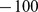, 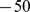, 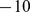, 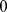, 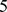, or 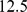 kb with respect to the start coordinate of the sequences. In total, this procedure generated 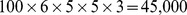 independent datasets for input into LDhelmet and LDhat.

#### Recurrent sweep model

In order to simulate datasets experiencing hard sweeps at random times and locations, we modified the software rsweep [45] to allow for a recombination hotspot rather than a constant recombination landscape. As above, we simulated datasets of 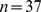 samples of 25 kb of sequence, this time under three realistic recurrent sweep models:
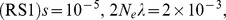


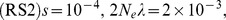


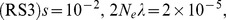
where 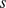 is the selection coefficient of new beneficial mutations and 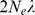 is the rate of fixation of beneficial mutations. In each case we took 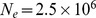. The first parameter combination is one of frequent, weak sweeps, and similar to the parameters estimated in [44]. The third combination is one of infrequent but stronger sweeps and similar to the parameters estimated in [45]. The second combination is intermediate between the two. Under a recurrent sweep model, selective sweeps occur at random times at a rate governed by 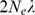 and at a location in the genome chosen uniformly at random. Sweeps both within the sequenced 25 kb and in flanking sequence can affect the observed data and are accounted for in the simulation software [45].

We considered 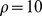 per kb for comparison with the single sweep model. As in the single sweep model, we simulated 100 datasets under each parameter combination, generating 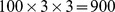 independent datasets for input into LDhelmet. As for the single sweep simulations, we ran LDhelmet for 250,000 iterations after 50,000 iterations of burn-in.

### Simulation study on the impact of demographic history

In order to simulate datasets that had been affected by a nonstandard demographic history, we used the software msHOT [74]. We investigated four realistic demographic histories:

(G1) Exponential growth at rate 100 initiated 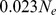 generations ago (a tenfold increase by the present time),

(G2) Exponential growth at rate 10 initiated 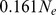 generations ago (a fivefold increase by the present time),

(B1) A bottleneck initiated 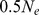 generations ago, with a transient reduction to size 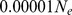 lasting 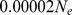 generations,

(B2) A bottleneck initiated 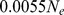 generations ago, with a transient reduction to size 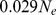 lasting 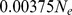 generations.

The first three models were proposed by Haddrill *et al.* [46] as reasonable fits to their (African) data, while the fourth is taken from [47] for a European population. We note that the precise demographic history of *D. melanogaster* populations remains poorly understood, and that these models simply serve as reasonable examples for investigating the robustness of our method. It is probable that there exist better fitting demographic models; indeed, Haddrill *et al.* ultimately favor their bottleneck model over any growth model.

We simulated 100 datasets under each model and under each of three recombination landscapes: a flat recombination rate of 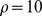 per kb except for a 2 kb-wide hotspot at the center of the sequence, of relative strength 1 (no hotspot), 10, or 50. This provided 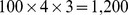 independent datasets in total. We also post-processed all outputs from the infinite-sites-based software to allow for a full quadra-allelic mutation model, using the mutation transition matrix 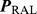 and the mutation rate 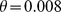 per bp. We ran LDhelmet for 250,000 iterations after 50,000 iterations of burn-in.

### Search for recombination hotspots

We used a conservative approach to identify candidate recombination hotspots. From the recombination maps for RAL and RG we first identified putative hotspots—regions in which the recombination rate exceeded ten times the mean for that chromosome arm, and which were greater than 500 bp in length. We discarded regions of length less than 500 bp on the grounds that such narrow peaks can be produced occasionally as spurious artifacts of the rjMCMC procedure.

To further filter the remaining candidate hotspots, we applied an independent method, sequenceLDhot [29], to the same data, in order to test for the presence of hotspots in these regions. The software uses a computationally-intensive importance sampling framework to construct likelihood ratios in sliding windows to evaluate the evidence for the presence of a hotspot in that window. To reduce computation time we focused on 50 kb regions centered on the autosomal putative hotspots. We modified sequenceLDhot's default parameters, which are tuned for interrogating human data, as follows. We used 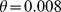 per site, and for the background recombination rate we used the estimated mean across the local 50 kb containing the hotspot of interest. We specified the software's grid for hotspot likelihoods to be in the range 10–100 times the background rate, and tested windows of 500 bp sliding in steps of 250 bp, using a composite likelihood comprising ten SNPs. Other parameters were unchanged. We reduced SNP density to be comparable to the data on which the software had been calibrated [29], by discarding sites with any missing alleles and singleton SNPs, though we obtained similar results without such a reduction (not shown). In constructing our final list of candidate hotspots, we retained only those which overlapped one of sequenceLDhot's ‘extended hotspot regions’, constructed conservatively from windows with a likelihood ratio greater than 10. To improve power in the search for hotspots, we included five additional lower coverage RG genomes in this analysis.

### Wavelet analysis

To put the recombination maps into a suitable time-series format, we used the (log-transformed) cumulative recombination rate across each 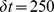 bp window. We found that this provided good resolution at high frequencies, with little further improvement using smaller bins. To facilitate a comparison between RAL and RG, we used the maps estimated from sample size 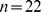 in both populations.

#### Continuous wavelet transform

Continuous wavelet transforms are useful for visualization purposes and for feature extraction, and the methods of *wavelet coherence* [54], [75] are based on them. All our plots of wavelet power are therefore based on continuous transforms, using software provided by [75] which convolves the data with the Morlet wavelet (parametrized by a frequency parameter 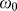; we take 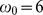). This choice of wavelet is reasonable because it is simple, widely used, and provides a sensible balance between time and frequency localization.

At large scales, the wavelet transform is influenced by data distant from a given position—possibly even outside the range of the data. The region of the time-frequency domain distorted by the consequent introduction of unwanted edge effects is said to be inside the *cone of influence*, which we define following [54] as the region in which wavelet power for a discontinuity at the edge drops by a factor of 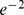. Results using the transform inside the cone of influence should be treated with caution.

To assess the significance of regions of the local wavelet power spectrum of high power, we assumed as a background power spectrum that of an autoregressive process of order 1 (AR(1)), whose underlying power spectrum is red noise. This serves as a simple, parametric way of positing an expected power spectrum for a dataset varying about some mean value and allowing for some autocorrelation. The distribution of the observed wavelet power taken with respect to the Morlet wavelet is, for each position and scale, then proportional to a 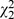 distribution under this model [54]. The autoregression parameter of the null model was estimated as that which best fit our observed data.

In order to identify regions of correlation of the wavelet transforms for the RAL and RG data, we performed a *wavelet coherence* analysis. Wavelet coherence is a (smoothed) measure of correlation which is computed as a function of both position and scale; we used the formulation given in [75]. To assess the significance of regions of high coherence, we again assume AR(1) models underlying the two datasets, and obtain critical coherence values using Monte Carlo simulation as described in [75] (with 1,000 Monte Carlo samples and 10 scales per octave).

#### Discrete wavelet transform

Because the scale index of a continuous wavelet transform varies continuously, coefficients at nearby scales encode similar information and a great deal of the transformed data is superfluous. On the other hand, the discrete wavelet transform provides a decomposition of the data into a minimal number of independent coefficients. It is therefore suitable for modeling purposes, since the transform is constructed so that variation in a signal at one scale is orthogonal to that at a different scale. Within the discrete set of scales chosen, those with important or significant variation can be identified unambiguously. In our linear model analyses we take the discrete wavelet transform based on the Haar wavelet, using methods and R scripts provided by [53]. Indeed, the paper by Spencer *et al.* [53] provides an excellent overview of the use of the discrete wavelet transforms in analyzing genomic data, and we refer the interested reader there for further details. Our analysis differs from theirs in several respects: (i) We analyzed five chromosome arms from two populations, giving ten datasets in total compared to their two, (ii) Since our data has much improved SNP density, we binned our data into 250 bp windows rather than 1 kb, giving a fourfold improved resolution, (iii) To control for the influence of local sequence quality, we used quality score information directly rather than read depth.

In addition to wavelet transforming the 250 bp-binned recombination map, we also binned and transformed a number of other genomic features: Diversity was computed as the mean, across pairs of samples within the population, of the fraction of sites that differed between the pair, out of a total of the number of sites for which both samples had data available. Divergence was computed as the diversity between the *D. melanogaster* and *D. simulans* reference sequences, which were available as part of a multiple sequence alignment along with the data from [24]. GC content was computed as the fraction of the total number of sequenced positions in the window (across all samples within the population) that were called as G or C. Gene content was computed for each window as the fraction of the window annotated as exonic; genome annotations were obtained from FlyBase (release 5.45, http://www.flybase.org [50]). Sequence quality scores were taken directly from the FASTQ files of the original data. Note that divergence and gene content data are the same for RAL and RG, explaining their identical power spectra in Figure 12.

## Supporting Information

Figure S1Comparison of the cumulative recombination maps of LDhelmet and LDhat for 25 datasets simulated under neutrality In each plot, different colors represent the cumulative recombination maps for different datasets. The datasets in these plots correspond to the same datasets used in Figure 1. The thick dashed line indicates the true cumulative recombination map for the given recombination landscape. The left and right columns show the estimated recombination maps of LDhelmet and LDhat, respectively, using the same block penalty of 50. (First Row) Each dataset was simulated with a constant recombination rate of 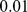 per bp. (Second Row) Each dataset was simulated with a hotspot of width 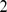 kb starting at location 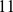 kb. The background recombination rate was 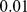 per bp, while the hotspot intensity was 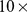 the background rate, i.e., 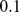 per bp. The cumulative maps are shown in their entirety, including potential edge effects.(EPS)

Figure S2Comparison of the cumulative recombination maps of LDhelmet and LDhat for 25 datasets simulated under strong positive selection. In each plot, different colors represent the results for different datasets. The datasets in these plots correspond to the same datasets used in Figure 3. The thick dashed line indicates the true cumulative recombination map for the given recombination landscape. The left and right columns show the estimated recombination maps of LDhelmet and LDhat, respectively, using the same block penalty of 50. In each simulation, the selected site was placed at position 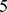 kb and the population-scaled selection coefficient was set to 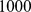. The fixation time of the selected site was 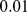 coalescent units in the past. The same scenarios of recombination patterns as in Figure 1 were considered: (First Row) with a constant recombination rate of 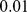 per bp, and (Second Row) with a hotspot of width 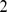 kb starting at location 11.5 kb. The background recombination rate was 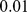 per bp, while the hotspot intensity was 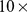 the background rate, i.e., 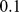 per bp. The cumulative maps are shown in their entirety, including potential edge effects.(EPS)

Figure S3Fisher's information for two-locus samples of size 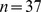 using lookup tables for 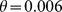 and under the infinite-sites assumption. The ancestral allele at each locus is assumed to be known.(EPS)

Figure S4 Distribution of recombination rates relative to transcription start sites. Plots for RAL (solid) and RG (dashed) of the average estimated recombination rate as a function of distance from the midpoint of the nearest transcription start site (TSS) to the left (negative x-axis) and to the right (positive x-axis) of every base. A 5-kb averaging window was used to smooth the estimates.
(EPS)

Figure S5 Local wavelet power spectrum of recombination rate variation in chromosome arm 2R. A power spectrum is shown for RAL and RG. Black contours denote regions of significant power at the 5% level, and the white contour denotes the cone of influence. Color scale is relative to a white-noise process with the same variance. The lower panels shows estimates of the corresponding genetic maps.
(EPS)

Figure S6 Local wavelet power spectrum of recombination rate variation in chromosome arm 3L. A power spectrum is shown for RAL and RG. Black contours denote regions of significant power at the 5% level, and the white contour denotes the cone of influence. Color scale is relative to a white-noise process with the same variance. The lower panels shows estimates of the corresponding genetic maps.
(EPS)

Figure S7 Local wavelet power spectrum of recombination rate variation in chromosome arm 3R. A power spectrum is shown for RAL and RG. Black contours denote regions of significant power at the 5% level, and the white contour denotes the cone of influence. Color scale is relative to a white-noise process with the same variance. The lower panels shows estimates of the corresponding genetic maps.
(EPS)

Figure S8 Local wavelet power spectrum of recombination rate variation in chromosome X. A power spectrum is shown for RAL and RG. Black contours denote regions of significant power at the 5% level, and the white contour denotes the cone of influence. Color scale is relative to a white-noise process with the same variance. The lower panels shows estimates of the corresponding genetic maps.
(EPS)

Figure S9 Pairwise correlation of detail wavelet coefficients of RAL and RG recombination maps for chromosome arms 2R, 3L, 3R, and X. Black circles denote Kendall's rank correlation between pairs of detail coefficients at each scale. Crosses denote the correlation that would be required for significance at the 1% level in a two-tailed test; red crosses are those scales at which the correlation is in fact significant.
(EPS)

Figure S10 Wavelet coherence analysis comparing RAL against RG for chromosome arms 2R, 3L, 3R, X. The cone of influence is shown in white.
(EPS)

Figure S11 Positive control for wavelet coherence analysis. (Left): Coherence plot for two independent estimates of the recombination map across chromosome arm 2L using the same (RG) dataset. (Right): The fraction of chromosome arm 2L with significantly high coherence at the 5% level, at each scale.
(EPS)

Figure S12 Global wavelet power spectrum and pairwise correlations of detail wavelet coefficients of RAL and RG data for chromosome arms 2R, 3L, 3R, and X. Diagonal plots show the global wavelet power spectrum of each feature of the RAL (blue) and RG (red) data. Off-diagonal plots show Kendall's rank correlation between pairs of detail coefficients at each scale, with respect to the wavelet decomposition of the two indicated features. Crosses denote the correlation that would be required for significance at the 1% level in a two-tailed test; red crosses are those scales at which the correlation is in fact significant. The lower left triangle and upper right triangle of plots correspond to RAL and RG, respectively.
(EPS)

Figure S13Linear model for wavelet transform of recombination map of chromosome arm 2R. (A) In a linear model for the detail coefficients of the wavelet transform of the recombination map of chromosome arm 2R, covariates are the detail coefficients of wavelet transforms of data quality, gene content, GC content, divergence, and diversity. Shown is the −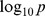-value of the regression coefficient at the given scale, as determined by a t-test. Colored boxes indicate significant relationships, with red positive and blue negative. Also shown in the adjusted 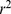. (B) As above, but with the recombination map of the other population as an additional covariate.(EPS)

Figure S14Linear model for wavelet transform of recombination map of chromosome arm 3L. (A) In a linear model for the detail coefficients of the wavelet transform of the recombination map of chromosome arm 3L, covariates are the detail coefficients of wavelet transforms of data quality, gene content, GC content, divergence, and diversity. Shown is the −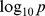-value of the regression coefficient at the given scale, as determined by a t-test. Colored boxes indicate significant relationships, with red positive and blue negative. Also shown in the adjusted 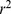. (B) As above, but with the recombination map of the other population as an additional covariate.(EPS)

Figure S15Linear model for wavelet transform of recombination map of chromosome arm 3R. (A) In a linear model for the detail coefficients of the wavelet transform of the recombination map of chromosome arm 3R, covariates are the detail coefficients of wavelet transforms of data quality, gene content, GC content, divergence, and diversity. Shown is the −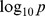-value of the regression coefficient at the given scale, as determined by a t-test. Colored boxes indicate significant relationships, with red positive and blue negative. Also shown in the adjusted 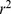. (B) As above, but with the recombination map of the other population as an additional covariate.(EPS)

Figure S16Linear model for wavelet transform of recombination map of chromosome X. (A) In a linear model for the detail coefficients of the wavelet transform of the recombination map of chromosome arm X, covariates are the detail coefficients of wavelet transforms of data quality, gene content, GC content, divergence, and diversity. Shown is the −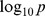-value of the regression coefficient at the given scale, as determined by a t-test. Colored boxes indicate significant relationships, with red positive and blue negative. Also shown in the adjusted 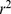. (B) As above, but with the recombination map of the other population as an additional covariate.(EPS)

Figure S17Plot of the average 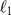-distance between the true and estimated recombination maps. Each plot shows the results averaged over 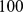 simulated datasets per block penalty for a given recombination landscape. In each simulation, we considered a 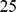 kb region with the background recombination rate of 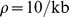. “no hotspot”: The true recombination map is constant. “hotspot 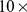
”: In the middle of the 25 kb region, the true recombination map has a hotspot of width 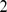 kb and intensity 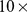 the background rate. “hotspot 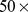
”: In the middle of the 25 kb region, the true recombination map has a hotspot of width 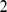 kb and intensity 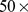 the background rate.(EPS)

Table S1Summary of comparison between LDhelmet and LDhat in the neutral case. Based on 100 simulated datasets for a 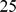 kb region. “No Hotspot” corresponds to the case of a constant recombination map, whereas “Hotspot 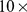
” corresponds to the case with a 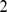 kb wide hotspot situated at the center of the region. The first row shows the regional average of 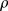 obtained by LDhelmet and LDhat, averaged over the 100 datasets. The second row shows the total rate in the hotspot region, averaged over the datasets. The third row shows the percentage of datasets for which the estimate had at least one false peak with height 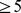 times the background rate. The fourth row shows the percentage of datasets for which the estimate had at least one false peak with height 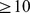 times the background rate. The fifth row shows the percentage absolute error of the estimated 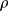 average outside the hotspot region from the true 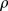 average outside the hotspot region. The true 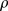 average outside the hotspot region is 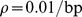. To account for edge effects, 2.5 kb from each end of the map were removed prior to computing the statistics.(PDF)

Table S2SNP densities (per kb) of neutral and single-sweep simulations. The mean, minimum, maximum and standard deviation of the SNP density for the datasets used in Tables S1 and S3. The simulations assumed a finite-sites, quadra-allelic mutation model, with mutation matrix 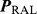 and 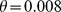, which is the effective population-scaled mutation rate adjusted for 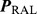 (see Estimation of mutation transition matrices).(PDF)

Table S3Summary of comparison between LDhelmet and LDhat in the case of single selective sweep. Based on 100 simulated datasets for a 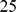 kb region. For each dataset, a selected site was placed at position 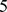 kb and the population-scaled selection coefficient was set to 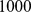. The fixation time of the selected site was 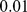 coalescent units in the past. The column and the row labels are the same as in Table S1. As for Table S1, 2.5 kb from each end of the map were removed prior to computing the statistics to account for edge effects.(PDF)

Table S4SNP densities (per kb) of recurrent-sweep and demography simulations. The statistics for each selection or demography scenario are merged over the three recombination landscapes (i.e., no hotspot, hotspot 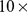 and hotspot 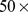). The simulations use 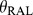 and as parameters. The third column shows the SNP density per kb across the hundred datasets, and the fourth column shows the standard deviation. For the definitions of the scenario names, refer to Simulation study on the impact of natural selection and Simulation study on the impact of demographic history of the main text. “Control” refers to a control dataset with constant population size and no selection.(PDF)

Table S5 SNP densities (per kb) of the North American (RAL) and the African (RG) *Drosophila* data.
(PDF)

Table S6Subsampling of real data. To assess the effect of subsampling individuals, we subsampled a 2 Mb excerpt from chromosome arm 2L for both the RAL and RG datasets. We performed subsampling four times, and each row is the average of the four subsampled datasets. The column labeled 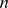 is the number of individuals in each subsample. The percentiles are given in the three rightmost columns. The results show that sample size has a slight positive bias, but does not impact estimates greatly.(PDF)

Table S7Thinned SNPs on RG dataset. To assess the effect of SNP density on the recombination rate inference, we thinned the SNPs on chromosome arm 2L and chromosome X of RG to the SNP density of RAL. The 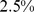, 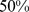 and 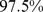 percentiles are shown for estimates. The number of SNPs in the original dataset and in the thinned dataset are shown in the fourth column. For chromosome arm 2L, the change in SNP density is negligible. For chromosome X, the difference in SNP density is significant. The results show that SNP density impacts the estimate, but not to the extent of the difference observed between RAL and RG on chromosome X.(PDF)

Table S8Exclusion of individuals with inversions. To assess the effect of inversions on the recombination rate estimate, we excluded individuals known to carry the given inversion, and performed inference on the remaining sample. 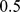 Mb was added to both ends of the region to eliminate possible edge effects.The 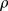 average is over the inversion region only. The column labeled Original gives the estimate using the entire sample. The column labeled Excluded gives the estimate excluding the individuals with the given inversion. The inversion region length and the number of individuals with the inversion are provided in the rightmost two columns.(PDF)

Table S9Running times (in seconds) for solving recursions and computing Padé coefficients. The second column is the time to solve the two-locus recursion described in Text S1 to compute the likelihood of a *single* value of 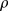 for all sample configurations of size 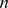. The third column is the time to compute 11 Padé coefficients for all sample configurations of size 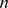. Recall that the recursion must be solved afresh for every value of 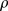 in the lookup table. On the other hand, the Padé coefficients are used to construct a rational function of 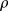 that approximates the likelihood; once the Padé coefficients are determined, evaluating the likelihood is instantaneous. A single 2.5 Ghz core was used in this benchmarking to provide representative estimates of the running time. However, note that both the recursion and Padé coefficient computations are highly parallelizable, which we exploit in the implementation of LDhelmet. Also note that the presence of missing data does not increase the running time for either computation.(PDF)

Text S1 Supplementary text on the two-locus recursion relation, Padé summation, and ancestral allele estimation.
(PDF)
