# Supplementary material for: Genome-Wide Fine-Scale Recombination Rate Variation in Drosophila melanogaster
Source: PLoS Genet. 2012 Dec 20;8(12):e1003090. doi: 10.1371/journal.pgen.1003090 (PMC3527307; doi:10.1371/journal.pgen.1003090)
Supplement: Table S1 — Summary of comparison between LDhelmet and LDhat in the neutral case. Based on 100 simulated datasets for a kb region. “No Hotspot” corresponds to the case of a constant recombination map, whereas “Hotspot ” corresponds to the case with a kb wide hotspot situated at the center of the region. The first row shows the regional average of obtained by LDhelmet and LDhat, averaged over the 100 datasets. The second row shows the total rate in the hotspot region, averaged over the datasets. The third row shows the percentage of datasets for which the estimate had at least one false peak with height times the background rate. The fourth row shows the percentage of datasets for which the estimate had at least one false peak with height times the background rate. The fifth row shows the percentage absolute error of the estimated average outside the hotspot region from the true average outside the hotspot region. The true average outside the hotspot region is . To account for edge effects, 2.5 kb from each end of the map were removed prior to computing the statistics. (PDF) [file pgen.1003090.s018.pdf]

Measure of Accuracy	No Hotspot			Hotspot 10×		
	True Value	LDhelmet	LDhat	True Value	LDhelmet	LDhat
ρ average (per bp)	0.01	0.0097	0.0109	0.0172	0.0184	0.0203
Total hotspot area	20.0	19.0	20.3	200.0	195.2	210.0
% with false peak $\geq 5\times$		5%	30%		4%	30%
% with false peak $\geq 10\times$		2%	21%		4%	21%
% abs. error outside hotspot region		14%	23%		15%	20%
