# Supplementary material for: Genome-Wide Fine-Scale Recombination Rate Variation in Drosophila melanogaster
Source: PLoS Genet. 2012 Dec 20;8(12):e1003090. doi: 10.1371/journal.pgen.1003090 (PMC3527307; doi:10.1371/journal.pgen.1003090)
Supplement: Table S2 — SNP densities (per kb) of neutral and single-sweep simulations. The mean, minimum, maximum and standard deviation of the SNP density for the datasets used in Tables S1 and S3. The simulations assumed a finite-sites, quadra-allelic mutation model, with mutation matrix and , which is the effective population-scaled mutation rate adjusted for (see Estimation of mutation transition matrices). (PDF) [file pgen.1003090.s019.pdf]

	Neutral		Single-Sweep Model	
	No Hotspot	Hotspot 10×	No Hotspot	Hotspot 10×
Mean	21.82	21.68	18.15	18.38
Min	18.32	17.40	14.84	14.68
Max	26.12	25.72	24.08	22.76
Std dev	1.71	1.38	1.64	0.61
