# Supplementary material for: Genome-Wide Fine-Scale Recombination Rate Variation in Drosophila melanogaster
Source: PLoS Genet. 2012 Dec 20;8(12):e1003090. doi: 10.1371/journal.pgen.1003090 (PMC3527307; doi:10.1371/journal.pgen.1003090)
Supplement: Table S3 — Summary of comparison between LDhelmet and LDhat in the case of single selective sweep. Based on 100 simulated datasets for a kb region. For each dataset, a selected site was placed at position kb and the population-scaled selection coefficient was set to . The fixation time of the selected site was coalescent units in the past. The column and the row labels are the same as in Table S1. As for Table S1, 2.5 kb from each end of the map were removed prior to computing the statistics to account for edge effects. (PDF) [file pgen.1003090.s020.pdf]

Measure of Accuracy	No Hotspot			Hotspot 10×		
	True Value	LDhelmet	LDhat	True Value	LDhelmet	LDhat
ρ average (per bp)	0.01	0.0079	0.0108	0.0172	0.0162	0.0220
Total hotspot area	20.0	14.7	15.4	200.0	169.8	224.6
% with false peak $\geq 5\times$		10%	42%		8%	34%
% with false peak $\geq 10\times$		6%	39%		5%	24%
% abs. error outside hotspot region		39%	58%		30%	56%
