# Supplementary material for: Genome-Wide Fine-Scale Recombination Rate Variation in Drosophila melanogaster
Source: PLoS Genet. 2012 Dec 20;8(12):e1003090. doi: 10.1371/journal.pgen.1003090 (PMC3527307; doi:10.1371/journal.pgen.1003090)
Supplement: Table S4 — SNP densities (per kb) of recurrent-sweep and demography simulations. The statistics for each selection or demography scenario are merged over the three recombination landscapes (i.e., no hotspot, hotspot and hotspot ). The simulations use and as parameters. The third column shows the SNP density per kb across the hundred datasets, and the fourth column shows the standard deviation. For the definitions of the scenario names, refer to Simulation study on the impact of natural selection and Simulation study on the impact of demographic history of the main text. “Control” refers to a control dataset with constant population size and no selection. (PDF) [file pgen.1003090.s021.pdf]

Simulation Type	Model	Mean	Std dev
Recurrent Sweeps	RS1	18.22	1.66
	RS2	4.10	1.05
	RS3	2.71	1.24
Demography	G1	12.86	1.07
	G2	15.85	1.24
	B1	13.84	2.78
	B2	5.53	2.14
Neutral	Control	22.51	1.49
