# Supplementary material for: Genome-Wide Fine-Scale Recombination Rate Variation in Drosophila melanogaster
Source: PLoS Genet. 2012 Dec 20;8(12):e1003090. doi: 10.1371/journal.pgen.1003090 (PMC3527307; doi:10.1371/journal.pgen.1003090)
Supplement: Table S5 — SNP densities (per kb) of the North American (RAL) and the African (RG) Drosophila data. (PDF) [file pgen.1003090.s022.pdf]

Chromosome Arm	RAL	RG
2L	24.54	25.49
2R	22.56	24.21
3L	22.29	25.20
3R	19.77	20.79
X	14.92	28.15
