# Supplementary material for: Genome-Wide Fine-Scale Recombination Rate Variation in Drosophila melanogaster
Source: PLoS Genet. 2012 Dec 20;8(12):e1003090. doi: 10.1371/journal.pgen.1003090 (PMC3527307; doi:10.1371/journal.pgen.1003090)
Supplement: Table S6 — Subsampling of real data. To assess the effect of subsampling individuals, we subsampled a 2 Mb excerpt from chromosome arm 2L for both the RAL and RG datasets. We performed subsampling four times, and each row is the average of the four subsampled datasets. The column labeled is the number of individuals in each subsample. The percentiles are given in the three rightmost columns. The results show that sample size has a slight positive bias, but does not impact estimates greatly. (PDF) [file pgen.1003090.s023.pdf]

		Percentile (ρ per kb)		
	n	2.5%	50%	97.5%
RAL	17	6.1	6.2	6.5
	27	7.2	7.3	7.4
	37	7.8	7.8	7.9
RG	12	8.1	8.4	9.2
	17	9.0	9.0	9.2
	22	9.2	9.3	9.4
