# Supplementary material for: Genome-Wide Fine-Scale Recombination Rate Variation in Drosophila melanogaster
Source: PLoS Genet. 2012 Dec 20;8(12):e1003090. doi: 10.1371/journal.pgen.1003090 (PMC3527307; doi:10.1371/journal.pgen.1003090)
Supplement: Table S7 — Thinned SNPs on RG dataset. To assess the effect of SNP density on the recombination rate inference, we thinned the SNPs on chromosome arm 2L and chromosome X of RG to the SNP density of RAL. The , and percentiles are shown for estimates. The number of SNPs in the original dataset and in the thinned dataset are shown in the fourth column. For chromosome arm 2L, the change in SNP density is negligible. For chromosome X, the difference in SNP density is significant. The results show that SNP density impacts the estimate, but not to the extent of the difference observed between RAL and RG on chromosome X. (PDF) [file pgen.1003090.s024.pdf]

| Dataset | Arm | Type | # SNPs | Percentile ($\rho$ per kb) | | |
|---|---|---|---|---|---|---|
| | | | | 2.5% | 50% | 97.5% |
| RG | 2L | Original | 586476 | 33.0 | 35.9 | 39.4 |
| | | Thinned | 564673 | 32.5 | 35.5 | 38.9 |
| | X | Original | 631205 | 110.0 | 121.4 | 134.1 |
| | | Thinned | 334647 | 97.5 | 106.8 | 117.4 |
