# Supplementary material for: Genome-Wide Fine-Scale Recombination Rate Variation in Drosophila melanogaster
Source: PLoS Genet. 2012 Dec 20;8(12):e1003090. doi: 10.1371/journal.pgen.1003090 (PMC3527307; doi:10.1371/journal.pgen.1003090)
Supplement: Table S8 — Exclusion of individuals with inversions. To assess the effect of inversions on the recombination rate estimate, we excluded individuals known to carry the given inversion, and performed inference on the remaining sample. Mb was added to both ends of the region to eliminate possible edge effects.The average is over the inversion region only. The column labeled Original gives the estimate using the entire sample. The column labeled Excluded gives the estimate excluding the individuals with the given inversion. The inversion region length and the number of individuals with the inversion are provided in the rightmost two columns. (PDF) [file pgen.1003090.s025.pdf]

Dataset	Arm	Inversion	**Original** ρ per kb	**Excluded** ρ per kb	Inversion length (Mb)	# with inversion
RAL	2L	2Lt	16.97	16.45	10.9	3
	2R	2RNS	17.34	16.66	4.9	2
	3R	3RK	11.80	11.39	14.4	1
	3R	3RMO	12.51	14.56	14.6	7
	3R	3RP	12.49	11.35	8.3	1
RG	2L	2Lt	54.44	50.80	10.9	2
	2R	2RNS	53.93	50.81	4.9	1
	3R	3RP	22.44	17.24	8.3	4
	X	1Be	106.26	103.21	1.8	3
