# Supplementary material for: Genome-Wide Fine-Scale Recombination Rate Variation in Drosophila melanogaster
Source: PLoS Genet. 2012 Dec 20;8(12):e1003090. doi: 10.1371/journal.pgen.1003090 (PMC3527307; doi:10.1371/journal.pgen.1003090)
Supplement: Table S9 — Running times (in seconds) for solving recursions and computing Padé coefficients. The second column is the time to solve the two-locus recursion described in Text S1 to compute the likelihood of a single value of for all sample configurations of size . The third column is the time to compute 11 Padé coefficients for all sample configurations of size . Recall that the recursion must be solved afresh for every value of in the lookup table. On the other hand, the Padé coefficients are used to construct a rational function of that approximates the likelihood; once the Padé coefficients are determined, evaluating the likelihood is instantaneous. A single 2.5 Ghz core was used in this benchmarking to provide representative estimates of the running time. However, note that both the recursion and Padé coefficient computations are highly parallelizable, which we exploit in the implementation of LDhelmet. Also note that the presence of missing data does not increase the running time for either computation. (PDF) [file pgen.1003090.s026.pdf]

Sample size n	Two-locus recursion (seconds)	Padé coefficients (seconds)
10	0.1	5
20	11	429
30	189	5271
40	1523	26405
50	7755	75704
