# Supplementary material for: Genome-Wide Fine-Scale Recombination Rate Variation in Drosophila melanogaster
Source: PLoS Genet. 2012 Dec 20;8(12):e1003090. doi: 10.1371/journal.pgen.1003090 (PMC3527307; doi:10.1371/journal.pgen.1003090)
Supplement: Text S1 — Supplementary text on the two-locus recursion relation, Padé summation, and ancestral allele estimation. (PDF) [file pgen.1003090.s027.pdf]

Supporting Information

Genome-wide fine-scale recombination rate variation in *Drosophila melanogaster*

Andrew H. Chan[1,*], Paul A. Jenkins[1,*], Yun S. Song[1,2,**]

[1] Computer Science Division, University of California, Berkeley, CA, USA
[2] Department of Statistics, University of California, Berkeley, CA, USA

[*] These authors contributed equally to this work
[**] Corresponding author e-mail: yss@cs.berkeley.edu

Text S1

Two-locus recursion relation

Suppose we sample n haplotypes, observing their alleles at each of two loci and obtaining configuration $\boldsymbol{n} = (\boldsymbol{a}, \boldsymbol{b}, \boldsymbol{c})$. Here $\boldsymbol{c} = (c_{ij})$ is a matrix of the counts of haplotypes for which both alleles were observed; c_{ij} is the number of haplotypes with allele i at the first locus and allele j at the second locus. We also allow for the possibility that a haplotype had data missing at one locus: $\boldsymbol{a} = (a_i)_{i=1\dots,K}$ is the vector of counts of haplotypes with allele i observed at the first locus and missing data at the second locus, and $\boldsymbol{b} = (b_j)_{j=1,\dots,L}$ is the vector of counts of haplotypes with allele j observed at the second locus and missing data at the first locus. Further, let:

$$a = \sum_{i=1}^{K} a_i, \quad c_{i\cdot} = \sum_{j=1}^{L} c_{ij}, \quad c = \sum_{i=1}^{K}\sum_{j=1}^{L} c_{ij},$$
$$b = \sum_{j=1}^{L} b_j, \quad c_{\cdot j} = \sum_{i=1}^{K} c_{ij}, \quad n = a + b + c.$$

The probability that, when we sample n haplotypes in some fixed order, we obtain a set consistent with configuration $\boldsymbol{n}$, is denoted by $q(\boldsymbol{n}; \theta_A, \theta_B, \rho)$. This probability is a function of θ_A, θ_B, and ρ: the mutation rates at the two loci, and the recombination rate between them. The respective mutation transition matrices at the two loci, which we denote $\boldsymbol{P}^A$ and $\boldsymbol{P}^B$, are fixed. A system of equations for $q(\boldsymbol{n}; \theta_A, \theta_B, \rho)$ is given in [1]. We denote by $q(\boldsymbol{n}, s_1, s_2; \theta_A, \theta_B, \rho)$ the joint probability of obtaining $\boldsymbol{n}$ with the events that there were precisely s_1 mutations in the history of the sample at the first locus and s_2 mutations in the history of the sample at the second locus. The corresponding system of equations for $q(\boldsymbol{n}, s_1, s_2; \theta_A, \theta_B, \rho)$ is:

$$[n(n-1) + \theta_A(a+c) + \theta_B(b+c) + \rho c]q((\boldsymbol{a}, \boldsymbol{b}, \boldsymbol{c}), s_1, s_2; \theta_A, \theta_B, \rho) =$$
$$\sum_{i=1}^{K} a_i(a_i - 1 + 2c_{i\cdot})q((\boldsymbol{a} - \boldsymbol{e}_i, \boldsymbol{b}, \boldsymbol{c}), s_1, s_2; \theta_A, \theta_B, \rho) + \sum_{j=1}^{L} b_j(b_j - 1 + 2c_{\cdot j})q((\boldsymbol{a}, \boldsymbol{b} - \boldsymbol{e}_j, \boldsymbol{c}), s_1, s_2; \theta_A, \theta_B, \rho)$$
$$+ \sum_{i=1}^{K}\sum_{j=1}^{L} [c_{ij}(c_{ij} - 1)q((\boldsymbol{a}, \boldsymbol{b}, \boldsymbol{c} - \boldsymbol{e}_{ij}), s_1, s_2; \theta_A, \theta_B, \rho) + 2a_i b_j q((\boldsymbol{a} - \boldsymbol{e}_i, \boldsymbol{b} - \boldsymbol{e}_j, \boldsymbol{c} + \boldsymbol{e}_{ij}), s_1, s_2; \theta_A, \theta_B, \rho)]$$
$$+ \theta_A \sum_{i=1}^{K} \Bigg[\sum_{j=1}^{L} c_{ij} \sum_{t=1}^{K} P_{ti}^{A} q((\boldsymbol{a}, \boldsymbol{b}, \boldsymbol{c} - \boldsymbol{e}_{ij} + \boldsymbol{e}_{tj}), s_1 - 1, s_2; \theta_A, \theta_B, \rho)$$

$$+ a_i \sum_{t=1}^{K} P^A_{ti} q((\boldsymbol{a} - \boldsymbol{e}_i + \boldsymbol{e}_t, \boldsymbol{b}, \boldsymbol{c}), s_1 - 1, s_2; \theta_A, \theta_B, \rho)\Bigg]$$

$$+ \theta_B \sum_{j=1}^{L} \Bigg[\sum_{i=1}^{K} c_{ij} \sum_{t=1}^{L} P^B_{tj} q((\boldsymbol{a}, \boldsymbol{b}, \boldsymbol{c} - \boldsymbol{e}_{ij} + \boldsymbol{e}_{it}), s_1, s_2 - 1; \theta_A, \theta_B, \rho)$$

$$+ b_j \sum_{t=1}^{L} P^B_{tj} q((\boldsymbol{a}, \boldsymbol{b} - \boldsymbol{e}_j + \boldsymbol{e}_t, \boldsymbol{c}), s_1, s_2 - 1; \theta_A, \theta_B, \rho)\Bigg]$$

$$+ \rho \sum_{i=1}^{K} \sum_{j=1}^{L} c_{ij} q((\boldsymbol{a} + \boldsymbol{e}_i, \boldsymbol{b} + \boldsymbol{e}_j, \boldsymbol{c} - \boldsymbol{e}_{ij}), s_1, s_2; \theta_A, \theta_B, \rho), \tag{1}$$

where $\boldsymbol{e}_{ij}$ is a unit matrix whose (i, j)th entry is one and the rest are zero. As before, we suppose that we know the identity of the ancestral alllele at each locus, say λ_A and λ_B at locus A and B, respectively. Then we replace the relevant instances of (1) with the following:

$$q((\boldsymbol{0}, \boldsymbol{b}, \boldsymbol{e}_{ij}), s_1, s_2; \theta_A, \theta_B, \rho) = \begin{cases} q((\boldsymbol{0}, \boldsymbol{b} + \boldsymbol{e}_j, \boldsymbol{0}), 0, s_2; \theta_A, \theta_B, \rho) & \text{if } i = \lambda_A \text{ and } s_1 = 0, \\ 0 & \text{otherwise,} \end{cases}$$

$$q((\boldsymbol{a}, \boldsymbol{0}, \boldsymbol{e}_{ij}), s_1, s_2; \theta_A, \theta_B, \rho) = \begin{cases} q((\boldsymbol{a} + \boldsymbol{e}_i, \boldsymbol{0}, \boldsymbol{0}), s_1, 0; \theta_A, \theta_B, \rho) & \text{if } j = \lambda_B \text{ and } s_2 = 0, \\ 0 & \text{otherwise,} \end{cases}$$

$$q((\boldsymbol{e}_i, \boldsymbol{0}, \boldsymbol{0}), s_1, s_2; \theta_A, \theta_B, \rho) = \begin{cases} 1 & \text{if } i = \lambda_A \text{ and } s_1 = s_2 = 0, \\ 0 & \text{otherwise,} \end{cases}$$

$$q((\boldsymbol{0}, \boldsymbol{e}_j, \boldsymbol{0}), s_1, s_2; \theta_A, \theta_B, \rho) = \begin{cases} 1 & \text{if } j = \lambda_B \text{ and } s_1 = s_2 = 0, \\ 0 & \text{otherwise.} \end{cases} \tag{2}$$

Padé summation

Modifications to the approach described in [2] are made, following from the boundary conditions given above. These can be converted into modifications of entries of the dynamic programming tables given in [2]. For example, using (2) we have that

$$\begin{aligned} q((\boldsymbol{a}, \boldsymbol{0}, \boldsymbol{e}_{i\lambda_B}), 1, 0; \theta_A, \theta_B, \rho) &= q((\boldsymbol{a} + \boldsymbol{e}_i, \boldsymbol{0}, \boldsymbol{0}), 1, 0; \theta_A, \theta_B, \rho) \\ &= q(\boldsymbol{a} + \boldsymbol{e}_i, 1; \theta_A) + \frac{0}{\rho} + \frac{0}{\rho^2} + \dots, \end{aligned}$$

where $q(\boldsymbol{a} + \boldsymbol{e}_i, 1; \theta_A)$ is the one-locus solution given by equation (3) in the main text. Notice that this expansion is in fact independent of ρ, from which it follows (by comparison with eq. (3.7) of [2]) that a number of entries in the dynamic programming tables are modified. For example, the second row in the dynamic programming table for the configuration $(\boldsymbol{a}, \boldsymbol{0}, \boldsymbol{e}_{i\lambda_B})$ is set to zero. Other boundary conditions may be interpreted in a similar fashion.

Ancestral allele estimation

Suppose we have one genomic sequence of *D. simulans* and n sequences of *D. melanogaster*. Let S represent the sequence of *D. simulans* and $M^{(k)}$ represent the sequence of the kth *D. melanogaster*, where S_l denotes the lth base of the sequence, and $S_{\hat{l}}$ represents the sequence with the exclusion of the lth base. Given $(S, M^{(k)})$, let $T_l^{(k)}$ be the time to the most recent common ancestor (TMRCA) at locus l; $f_l^{(k)}(t \mid M_{\hat{l}}, S_{\hat{l}})$ be the density of the TMRCA conditioned on both their sequences but *excluding* the lth locus; and $A_l^{(k)}$ be the ancestral allele at the lth locus, i.e., the allele of the most recent common ancestor (MRCA).

To compute the distribution on the ancestral allele at the lth locus conditioned on $M^{(k)}$ and S, we use

Bayes' theorem to obtain

$$\begin{aligned}
&\mathbb{P}(A_l^{(k)} = i \mid M^{(k)}, S) \\
&= \frac{\int_0^\infty p(A_l^{(k)} = i, M^{(k)}, S, T_l^{(k)} = t)dt}{\mathbb{P}(M^{(k)}, S)} \\
&= \frac{\int_0^\infty \mathbb{P}(M_l^{(k)}, S_l^{(k)} \mid A_l^{(k)} = i, T_l^{(k)})p(A_l^{(k)} = i, T_l^{(k)} = t)dt}{\mathbb{P}(M^{(k)}, S)} \\
&= \frac{\int_0^\infty \mathbb{P}(M_l^{(k)} \mid A_l^{(k)} = i, T_l^{(k)} = t)\mathbb{P}(S_l \mid A_l^{(k)} = i, T_l^{(k)} = t)\mathbb{P}(A_l^{(k)} = i)f_l^{(k)}(t \mid M_{\hat{l}}^{(k)}, S_{\hat{l}})dt}{\sum_j \int_0^\infty \mathbb{P}(M_l^{(k)} \mid A_l^{(k)} = j, T_l^{(k)} = t)\mathbb{P}(S_l \mid A_l^{(k)} = j, T_l^{(k)} = t)\mathbb{P}(A_l^{(k)} = j)f_l^{(k)}(t \mid M_{\hat{l}}^{(k)}, S_{\hat{l}})dt}. \qquad (3)
\end{aligned}$$

In equation (3), the prior on the ancestral allele at locus l, $\mathbb{P}(A_l^{(k)} = i)$, is given by the stationary distribution of the allele frequencies from the mutation matrix $\boldsymbol{P}$. (In the above, p denotes a joint probability of discrete events together with the density for $T_l^{(k)}$.) The density on the TMRCA, $f_l^{(k)}(t \mid M_{\hat{l}}^{(k)}, S_{\hat{l}})$, is estimated using Li & Durbin's `psmc` [3]. In practice, we use `psmc` to compute $f_l^{(k)}(t \mid M^{(k)}, S)$ and assume $f_l^{(k)}(t \mid M^{(k)}, S) \approx f_l^{(k)}(t \mid M_{\hat{l}}^{(k)}, S_{\hat{l}})$.

The remaining two probabilities, $\mathbb{P}(M_l^{(k)} \mid A_l^{(k)} = i, T_l^{(k)} = t)$ and $\mathbb{P}(S_l \mid A_l^{(k)} = i, T_l^{(k)} = t)$, are computed as follows. For the computation of $\mathbb{P}(M_l^{(k)} \mid A_l^{(k)} = i, T_l^{(k)} = t)$, let $\boldsymbol{P} = (P_{ij})$ denote the mutation matrix, and let $r_l^{(k)}$ specify the number of mutations that have occurred at the lth locus of the kth *D. melanogaster* sequence during time $T_l^{(k)}$. Then we have

$$\begin{aligned}
\mathbb{P}(M_l^{(k)} = j \mid A_l^{(k)} = i, T_l^{(k)} = t) &= \sum_{s=0}^{\infty} \mathbb{P}(r_l^{(k)} = s \mid T_l^{(k)} = t)(\boldsymbol{P}^s)_{ij} \\
&= \sum_{s=0}^{\infty} \left(\frac{\theta t}{2}\right)^s \frac{e^{-\theta t/2}}{s!}(\boldsymbol{P}^s)_{ij} \\
&= \sum_{s=0}^{\infty} \left[\left(\frac{\theta t}{2}\boldsymbol{P}\right)^s\right]_{ij} \frac{e^{-\theta t/2}}{s!} \\
&= \left[e^{\frac{\theta t}{2}(\boldsymbol{P}-\mathbf{I})}\right]_{ij},
\end{aligned}$$

where $\mathbf{I}$ is the identity matrix with the same dimensions as $\boldsymbol{P}$. The computation for $\boldsymbol{P}(S_l \mid A_l^{(k)} = j, T_l^{(k)} = t)$ is analogous.

After computing $\mathbb{P}(A_l^{(k)} = i \mid M^{(k)}, S)$ for every k and given l, we heuristically aggregate these pairwise probabilities to estimate $\mathbb{P}(A_l^{(k)} = i \mid M^{(1)}, \ldots, M^{(n)}, S)$ as follows. Let $\bar{t}_l^{(k)}$ be the posterior mean of $f_l^{(k)}(t \mid M^{(k)}, S)$, i.e.:

$$\bar{t}_l^{(k)} = \int_0^\infty t f_l^{(k)}(t \mid M^{(k)}, S)dt,$$

and define $\tau_l = \max_k \bar{t}_l^{(k)}$. We approximate $\mathbb{P}(A_l^{(k)} = i \mid M^{(1)}, \ldots, M^{(n)}, S)$ as

$$\mathbb{P}(A_l^{(k)} = i \mid M^{(1)}, \ldots, M^{(n)}, S) \approx \frac{\sum_{k=1}^n \mathbb{P}(A_l^{(k)} = i \mid M^{(k)}, S)f_l^{(k)}(\tau_l \mid M_{\hat{l}}^{(k)}, S_{\hat{l}})}{\sum_j \sum_{k=1}^n \mathbb{P}(A_l^{(k)} = j \mid M^{(k)}, S)f_l^{(k)}(\tau_l \mid M_{\hat{l}}^{(k)}, S_{\hat{l}})},$$

which is a weighted average of $\mathbb{P}(A_l^{(k)} = i \mid M^{(k)}, S)$ over k, weighted by the density of the TMRCA evaluated at τ_l for each k. This averaging ought to mitigate effects such as genotyping errors and incomplete lineage sorting in individual *D. melanogaster* genomes.

References

1. Jenkins PA, Song YS (2009) Closed-form two-locus sampling distributions: accuracy and universality. Genetics 183: 1087–1103.

2. Jenkins PA, Song YS (2012) Padé approximants and exact two-locus sampling distributions. Annals of Applied Probability 22: 576–607.

3. Li H, Durbin R (2011) Inference of human population history from individual whole-genome sequences. Nature 475: 493–496.
